# A randomised controlled trial of treatments of childhood anxiety disorder in the context of maternal anxiety disorder: clinical and cost‐effectiveness outcomes

**DOI:** 10.1111/jcpp.13089

**Published:** 2019-07-31

**Authors:** Cathy Creswell, Mara Violato, Susan Cruddace, Stephen Gerry, Lynne Murray, Roz Shafran, Alan Stein, Lucy Willetts, Emma McIntosh, Peter J. Cooper

**Affiliations:** ^1^ Department of Psychiatry University of Oxford Oxford UK; ^2^ Department of Experimental Psychology University of Oxford Oxford UK; ^3^ School of Psychology and Clinical Language Sciences University of Reading Reading UK; ^4^ Nuffield Department of Population Health University of Oxford Oxford UK; ^5^ Nuffield Department of Orthopaedics, Rheumatology & Musculoskeletal Science University of Oxford Oxford UK; ^6^ Population, Policy and Practice UCL Great Ormond Street Institute of Child Health London UK; ^7^ MRC/Wits Rural Public Health and Health Transitions Research Unit (Agincourt) University of the Witwatersrand Johannesburg South Africa; ^8^ Berkshire Healthcare NHS Foundation Trust Berkshire UK; ^9^ Institute of Health and Wellbeing University of Glasgow Glasgow UK

**Keywords:** Child, anxiety, mother, parent–child interaction, cognitive behaviour therapy

## Abstract

**Background:**

This study evaluated whether clinical and economic outcomes from CBT for child anxiety disorders in the context of maternal anxiety disorders are improved by adding treatment focused on (a) maternal anxiety disorders or (b) mother–child interactions.

**Methods:**

Two hundred and eleven children (7–12 years, 85% White British, 52% female) with a primary anxiety disorder, whose mothers also had a current anxiety disorder, were randomised to receive (a) child‐focused CBT with nonspecific control interventions (CCBT+Con), (b) CCBT with CBT for the maternal anxiety disorder (CCBT+MCBT), or (c) CCBT with an intervention targeting the mother–child interaction (CCBT+MCI). A cost‐utility analysis from a societal perspective was conducted using mother/child combined quality‐adjusted life years (QALYs). [Trial registration: https://doi.org/10.1186/isrctn19762288].

**Results:**

MCBT was associated with immediate reductions in maternal anxiety compared to the nonspecific control; however, after children had also received CCBT, maternal outcomes in the CCBT+MCI and CCBT+Con arms improved and CCBT+MCBT was no longer superior. Neither CCBT+MCBT nor CCBT+MCI conferred a benefit over CCBT+Con in terms of child anxiety disorder diagnoses post‐treatment [primary outcome] (*adj RR*: 1.22 (95% CI: 0.88, 1.67), *p *=* *.23; *adj RR*: 1.21 (95% CI: 0.88, 1.65), *p *=* *.24, respectively) or global improvement ratings (*adj RR*: 1.25 (95% CI: 0.99, 1.57), *p *=* *.06; *adj RR*: 1.18 (95% CI: 0.93, 1.50), *p *=* *.17) or six and 12 months later. No significant differences between the groups were found on the main economic outcome measures (child/mother combined QALY mean difference: CCBT+MCBT vs. CCBT+Con: −0.04 (95% CI: −0.12, 0.04), *p *=* *.29; CCBT+MCI vs. CCBT+Con: 0.02 (95% CI: −0.05, −0.09), *p *=* *.54). CCBT+MCI was associated with nonsignificantly higher costs than CCBT (mean difference: £154 (95% CI: −£1,239, £1,547), *p *=* *.83) but, when taking into account sampling uncertainty, it may be cost‐effective compared with CCBT alone.

**Conclusions:**

Good outcomes were achieved for children and their mothers across treatment arms. There was no evidence of significant clinical benefit from supplementing CCBT with either CBT for the maternal anxiety disorder or treatment focussed on mother–child interactions, but the addition of MCI (and not MCBT) may be cost‐effective.

## Introduction

Anxiety disorders are among the most common psychological disorders in childhood affecting 2.6%–5.2% of children under the age of 12 years (e.g. Costello, Egger, & Angold, 2004). They negatively affect children's functioning (Essau, Conradt, & Petermann, 2000), raise the risk for disorders in adolescence and adulthood (Woodward & Fergusson, 2001), and carry a substantial health and social cost (Bodden, Dirksen, & Bogels, 2008). There is considerable evidence for the efficacy of cognitive behaviour therapy (CBT) for these disorders, with 59% of anxious children no longer meeting criteria for an anxiety disorder following CBT (James, James, Cowdrey, Soler, & Choke, 2013). However, where a parent has an anxiety disorder, rates of child recovery have been found to be as little as half compared to when there is no parental anxiety disorder (Bodden, Bögels et al., 2008; Hudson et al., 2014). Two studies to date have examined whether specifically targeting elevated parental anxiety might benefit child treatment outcome (with treatments delivered in group formats). One of these studies reported a positive impact on anxious children of parents with high trait anxiety (Cobham, Dadds, & Spence, 1998), at least in the short term, while the other reported no benefit for anxious children of parents with a current anxiety disorder (Hudson et al., 2014). Notably, both of these studies administered relatively brief treatments for parental anxiety (4 and 3.75 hr, respectively) and neither brought about significant reductions in parental anxiety. The question therefore remains open whether successful treatment of parental anxiety might benefit child outcome.

It is possible that targeting parent–child interactions that are likely to interfere with the child's treatment – that is reducing parent cognitions and behaviours that limit the child's autonomy and promote the child's focus on threat (Creswell, Murray, Stacey, & Cooper, 2011; Hudson & Rapee, 2004) – may be of benefit for anxious children in the context of parental anxiety disorder (Settipani, O'Neil, Podell, Beidas, & Kendall, 2013). Indeed, parental cognitions characterised by expectations that the child will be frightened and feel out of control in the face of a challenge and behaviours characterised by intrusiveness and modelling of fear have been found to occur more commonly in anxious compared to nonanxious parents of children with anxiety disorders (Creswell, Apetroaia, Murray, & Cooper, 2013; Moore, Whaley, & Sigman, 2004).

In view of the roles of parental anxiety as well as parenting cognitions and behaviour in maintaining child anxiety, the current study tested the hypothesis that clinical outcomes for child anxiety disorders presenting in the context of parental anxiety disorders will be improved by supplementing individual child CBT with (a) treatment of parental anxiety disorders or (b) treatment focused on parent–child interactions.

This study also evaluated whether supplementing CBT with these adjunct treatments represents good value for money. The few published cost‐effectiveness analyses of the treatment of child anxiety mainly concern the value of child CBT compared with a wider family approach (Bodden, Bögels et al., 2008; Bodden, Dirksen, & Bogels, 2008) or with a parent‐directed treatment for child anxiety (Simon, Dirksen, Bögels, & Bodden, 2012). There is, however, a paucity of evidence on the short‐term and long‐term cost‐effectiveness of alternative configurations of treatment types involving maternal and child treatment combinations, which are likely to be of particular relevance when maternal anxiety disorder is present. Furthermore, no economic evaluations of the treatment of child anxiety have taken into account both the direct effects to the child and ‘spillover’ effects, for example, to the parents and wider society. For outcomes, this is achieved by using quality‐adjusted life years (QALY), a generic measure of outcome that can be aggregated across individuals (Weinstein, Torrance, & McGuire, 2009) and allows for examination of both direct treatment and spillover effects (Tilford et al., 2015). For costs, this is achieved by measuring broader impacts on education and employment. Aggregating direct and spillover effects may alter cost‐effectiveness profiles, a particularly appropriate strategy when the economic evaluation adopts a societal perspective. Indeed, recommendations from a recent review of family‐based therapies suggest that future research in this field should include such spillover effects to the rest of the family as well as consider cost and effectiveness over a longer time horizon (Tubeuf & Guthmuller, 2017). Further, few evaluations to date have formally aggregated QALYs as a means of capturing spillover effects. The current study therefore tested the value for money of the two supplementary interventions, considering spillover effects, societal impacts and including a one‐year follow‐up.

## Methods

### Participants

Participants comprised 211 children, aged 7–12 years (mean age = 10.22, *SD* = 1.58), with a current anxiety disorder, together with their mothers, who also had a current anxiety disorder. The study was powered to provide 90% power at the 5% (two‐sided) significance level to detect a 30% difference in the primary outcome – that is the proportion of children who recovered from their primary anxiety disorder at the post‐treatment assessment in the CCBT+MCI or CCBT+MCBT arms compared to the CCBT+Con arm. The estimated remission rate for the CCBT+Con group was 40% (based on Cobham et al., 1998). A difference of 30% in the proportion of anxiety‐free children following completion of the treatment was considered to be the minimum that would be clinically worthwhile taking into account the increased resources required and change to service delivery that would be required if either of these interventions was found to be effective and implemented in practice. The sample size was defined on the basis of two independent trials as recommended by Machin, Campbell, Fayers, and Pinol (1997). The required sample size of 56 children per group was increased to allow for an estimated 20% loss to follow‐up.

The study focussed on mothers as (a) intergenerational associations for anxiety disorders are most commonly found between mothers and their children (Cooper, Fearn, Willetts, Seabrook, & Parkinson, 2006), (b) mothers are most commonly the primary caregivers in the study region, and (c) maternal and paternal behaviours may differ in how they are associated with childhood anxiety (Bögels & Perotti, 2011).

**Figure 1:**
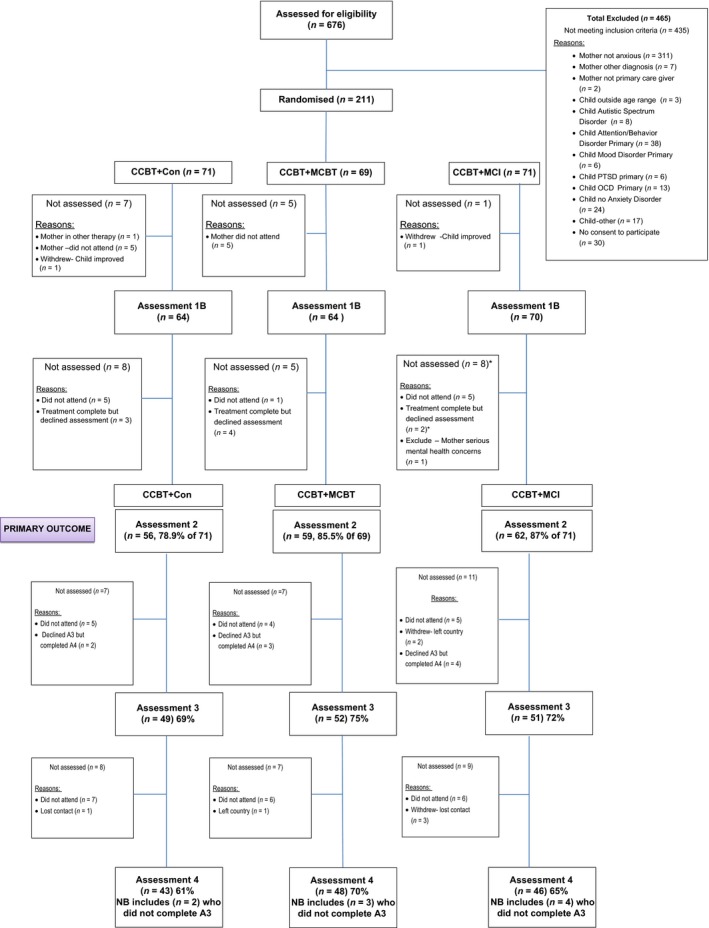
Consort diagram. CCBT+Con, child‐focused cognitive behaviour therapy + nonspecific control interventions; CCBT+MCBT, CCBT + CBT to target maternal anxiety disorder; CCBT+MCI, CCBT + intervention to target the mother–child interaction [Colour figure can be viewed at http://www.wileyonlinelibrary.com]

Participants were recruited between June 2008 and May 2011, with the last follow‐up assessment in February 2013. Participating children were all referred to a university research clinic for treatment by a health or educational professional. The inclusion criteria were (a) the child must (i) be aged 7–12 years; (ii) have a current primary diagnosis of a DSM‐IV anxiety disorder (if specific phobia, this must be comorbid with another anxiety disorder); and (b) the child's mother must (i) be the primary carer; and (ii) meet criteria for a current DSM‐IV anxiety disorder. The exclusion criteria were (i) significant physical or intellectual impairment (including autistic spectrum disorders) in the child or mother; (ii) current prescription of psychotropic medication for the child or mother that had not been at a stable dose for at least one month and without agreement to maintain that dose throughout the study; and (iii) the child had previously received six or more sessions of CCBT for an anxiety disorder. Participating children and mothers were given a complete description of the study orally and in writing before giving written informed assent/consent. A total of 676 children were assessed for eligibility; 435 families did not meet the inclusion criteria and 30 declined to participate (see Figure 1).

### Procedure

The study was approved by the Berkshire Research Ethics Committee (07/H0505/156) and the University of Reading Research Ethics Committee (07/48). Participants were randomised to one of three treatment arms: (a) child cognitive behaviour therapy plus control interventions to balance for therapist time (CCBT+Con) (see below); (b) CCBT plus cognitive behaviour therapy for maternal anxiety disorder (CCBT+MCBT); or (c) CCBT plus treatment focused on the mother–child interaction (CCBT+MCI). Each of the three arms included nonspecific therapeutic interventions to ensure treatment arms were balanced for therapist contact with both children and mothers (see Table 1).

**Table 1:** Overview of design

	**CCBT+MCBT**	**CCBT+MCI**	**CCBT+Con**
Assessment 1 Pretreatment	Diagnostic assessment (mother and child) + laboratory observation of mother–child interaction		
**Treatment 1** **(number of sessions)**	**MCBT (8)**	**NDC (2)**	**NDC (8)**
Assessment 1B Mid‐treatment	Diagnostic assessment (mother and child)		
**Treatment 2** **(number of sessions)**	**CCBT (8) + HLC (Mother:2; Child + mother: 2)**	**CCBT (8) + MCI (Mother:8; Child + Mother: 2)**	**CCBT (8) + HLC (Mother:2; Child + mother: 2)**
Assessment 2 Post‐treatment	Diagnostic assessment (mother and child) + laboratory observation of mother–child interaction		
Assessment 3 6 months post‐treatment	Diagnostic assessment (child)		
Assessment 4 12 months post‐treatment	Diagnostic assessment (child)		
**Total therapy sessions**	**Mother: 10** **Child: 2** **Child + Mother: 2**	**Mother: 10** **Child: 2** **Child + Mother: 2**	**Mother: 10** **Child: 2** **Child + Mother: 2**

CCBT, child‐focused cognitive behaviour therapy; CCBT‐Con, child‐focused cognitive behaviour therapy plus nonspecific control interventions; MCBT, cognitive behaviour therapy targeting maternal anxiety diagnoses; NDC, nondirective counselling for mothers; HLC, Healthy Living Control; MCI, mother–child interaction focussed treatment.

Randomisation was performed externally at the Centre for Statistics in Medicine (University of Oxford**)** on receipt of anonymised participant information by fax. Patients were randomised with a 1:1:1 ratio, with minimisation for child age and gender, type of child anxiety disorder, and baseline severity of both child and maternal primary anxiety disorder. These minimisation variables were selected as they have been most frequently (albeit not consistently) identified as predictors of response to treatment for child anxiety disorders (Ginsburg et al., 2011; Hudson et al., 2015). The minimisation algorithm included a 20% random element.

Assessments of maternal anxiety disorder and parenting were made before and immediately following the interventions. Assessments of child anxiety disorder status and severity were conducted before and following treatment, as well as at six and 12 months following treatment. Different research staff assessed the mother and the child. Assessments of maternal and child health‐related quality of life were made on five occasions: before treatment (Assessment 1), mid‐way through treatment (1B), immediately post‐treatment (2), and at the six (3)‐ and 12‐month (4) follow‐up. Data on the use of other health and social care resources, as well as non‐NHS cost‐generating (e.g. educational) services and days off school or work, were obtained from parent report. All assessors were blind to treatment allocation. The trial was preregistered as follows https://doi.org/10.1186/isrctn19762288. The protocol is provided in Appendix S1.

### Measures

#### Structured diagnostic interviews with children and parents

Children were assigned diagnoses on the basis of the Anxiety Disorders Interview Schedule for DSM‐IV for Children – Child and Parent Versions (ADIS‐C/P; Silverman & Albano, 1996). The presence or absence of a current maternal anxiety disorder was assigned on the basis of the Anxiety Disorders Interview Schedule for DSM‐IV (ADIS‐IV; DiNardo, Barlow, & Brown, 1994). For the ADIS‐C/P overall diagnoses and clinical severity ratings (CSRs) were assigned if the child met diagnostic criteria on the basis of either child or parent report. Those with a CSR of 4 or more (at least moderate psychopathology) were considered to meet diagnostic criteria. Assessors were thoroughly trained to ensure high levels of reliability (presence/absence of child diagnosis *κ = *.98 (child report), .98 (mother report); CSR intraclass correlation = .99 (child report), .99 (mother report); presence/absence of maternal diagnosis *κ = *.97; CSR intraclass correlation = .99). The primary outcome derived was absence of the child's primary (most impairing) anxiety disorder; secondary outcomes derived from the ADIS‐c/p were absence of all anxiety disorder diagnoses and CSR of the primary anxiety disorder, as prespecified in the trial protocol and statistical analysis plan (see Appendices S1 and S2).

#### Clinical Global Impressions: Improvement (CGI‐I)

Overall improvement in child anxiety was assessed using the Clinical Global Impression – Improvement scale (CGI‐I), a seven‐point scale from 1 = very much improved to 7 = very much worse; scores of 1 and 2 are accepted to represent treatment success (Guy, 1976). Overall mean inter‐rater reliability for the assessment team was high (ICC* *=* *.96).

*Symptoms of anxiety and comorbid difficulties* were also assessed as secondary outcomes using the Spence Child Anxiety Scale (child, parent, teacher report), Child Anxiety Impact Scale (child, parent report), Short Mood and Feelings Questionnaire (child, parent report), conduct problems subscale of the Strengths and Difficulties Questionnaire (child, parent, teacher report) and a teacher report measure of Child Adjustment to School. Descriptions of measures and outcomes are provided in Table S1.

#### Assessment of parenting and parental expectations

In order to establish whether the intervention that targeted mother–child interactions did successfully alter maternal responses, we conducted laboratory observations under conditions of mild social, performance and physical challenge (Creswell et al., 2013). The social threat task involved the child preparing and delivering a speech, with their mother's support, to a research assistant with a hand‐held video camera. The performance task involved the child attempting difficult tangram puzzles (following the procedure of Hudson & Rapee, 2001). The physical threat task required children to investigate the content of four chambers within a mysterious ‘black box’. To account for prior experience, the assessment was modified at the post‐treatment assessment point; in the social stress task, the child was required to present to a panel rather than a single research assistant, the tangram puzzles were more difficult, and the black box was accompanied by sound effects (i.e. rustling/scratching).

Observers who were blind to treatment arm coded parental behaviours on scales developed by Murray et al. (2012) and adapted by Creswell et al. (2013) to be suitable for children aged 7–12 years. Ratings were given for each minute of the interaction on 5‐point scales (1 = none, 5 = pervasive/strong). Since interactions varied in duration, total scores across tasks reflected the sum of the mean scores for each task. The following behaviours were considered: maternal expressed anxiety, overprotection, intrusiveness and positivity (warmth and encouragement). For each coder, in each task, a second coder independently scored a random sample of 25 videotapes. Intraclass correlations showed acceptable agreement across all indices (range .60–1.00; mean: .86).

Maternal expectations were assessed before initiating the challenge tasks (Creswell et al., 2013). Immediately after receiving the instructions for each task, mothers were taken to a separate room and asked to provide ratings regarding (a) how their child would feel about doing the task (0 = not scared at all, 10 = extremely scared); and (b) how much their child could do about how the task went (0 = nothing at all, 10 = a lot). Ratings were combined across the three tasks to represent their expectations across a range of challenge contexts.

#### Health‐related quality of life

Mother and child health‐related quality of life (HRQoL) was measured using responses to the EuroQol EQ‐5D‐3L (Dolan, Gudex, Kind, & Williams, 1995) and EQ‐5D‐Youth version (Wille et al., 2010) instruments, respectively. Responses for mother and child at each time point were converted into utility weights using the UK population tariff (Dolan et al., 1995). Total quality‐adjusted life years (QALYs) were calculated for each mother and child, using the area under the curve approach after linear interpolation between the time points. Mother–child dyad QALYs were obtained by additively combining individual mother and child QALYs (Weinstein et al., 2009).

A broad, societal perspective (including parental productivity and school impacts) was adopted in assessing resource use and costs. In addition to the intervention costs (captured through therapists’ logs completed at each therapy session), values were attached for measured school absence, time off work, and lost leisure time (for mothers), use of non‐NHS services (e.g. educational services) and personal costs of medications, all of which was captured through parent diaries. An NHS perspective was also adopted as per NICE guidance (NICE, 2013).

### Treatment

#### Child Cognitive Behaviour Therapy (CCBT)

All children received eight‐weekly one‐hour sessions of individual CBT. Treatment was delivered by one of seven qualified clinical psychologists or cognitive behaviour therapists, following a manual based on the ‘Cool Kids’ (Lyneham, Abbott, Wignall, & Rapee, 2003) and ‘Coping Cat’ (Kendall & Hedtke, 2006) programmes (both of which have established efficacy; Hudson et al., 2009; Walkup et al., 2008). While these manuals include more than eight treatment sessions, there is evidence of good outcomes from similar CBT content for child anxiety disorders delivered in eight hours or less (e.g. Gallagher, Rabian, & McCloskey, 2004; Ginsburg & Drake, 2002). CCBT was supervised by a highly experienced, clinical psychologist and accredited cognitive behaviour therapist. Treatment focused on helping children to identify and challenge negative thinking styles, gradually increasing exposure to feared stimuli and developing problem‐solving skills. Mothers were included at the beginning and end of each session to share progress and feedback. As described in Appendix S3, CCBT treatment adherence was equivalent across treatment arms.

#### CBT for maternal anxiety disorder (MCBT)

MCBT consisted of eight one‐hour weekly sessions delivered by one of five qualified clinical psychologists or cognitive behaviour therapists (all supervised by a highly experienced, clinical psychologist and accredited cognitive behaviour therapist) over 8 weeks. Given that mothers presented with a range of anxiety disorders and given evidence for the efficacy of transdiagnostic CBT treatments for anxiety disorders (Newby, McKinnon, Kuyken, Gilbody, & Dalgleish, 2015), we followed a manualised transdiagnostic treatment for adult disorders which aimed to reverse the putative cognitive behavioural maintaining mechanisms identified through individual formulation This manual has not previously been systematically evaluated; however, promising results were obtained in a case series evaluation (McManus, Clark, Muse, & Shafran, 2015).

In the treatment arms that did not involve MCBT, mothers received nondirective counselling (NDC), a supportive individual intervention that was not focussed specifically on reducing symptoms of anxiety. NDC was provided by one of four qualified counsellors (supervised by a highly experienced counsellor/psychotherapist) following the manual of Borkovec and Costello (1993). As shown in Appendix S3, the MCBT and NDC were clearly distinct. MCBT and NDC were delivered first, before the delivery of CCBT (see Table 1).

#### Mother–Child Interaction treatment (MCI)

The MCI intervention consisted of 10 sessions delivered over 8 weeks by one of five qualified clinical psychologists or cognitive behaviour therapists (supervised by an experienced clinical psychologist): eight sessions were with the mother alone and two were with the mother and child together. This was a novel intervention designed to target potentially anxiogenic features of the mother–child relationship. Specifically, it aimed to enhance maternal autonomy promoting cognitions (such as confidence in the child's ability to face challenge) and behaviours, and reduce potentially anxiogenic behaviours. This was achieved through a combination of specific strategies from existing family interventions for childhood anxiety (Lyneham et al., 2003) with the addition of video‐feedback techniques developed and piloted by the trial investigators. The two joint mother and child sessions involved the mother and child completing structured tasks that were video‐recorded for reference in later sessions.

To balance therapist contact, sessions that focused on the promotion of a healthy lifestyle (Healthy Living Control, HLC) were delivered in the treatment arms that did not receive the MCI intervention. This intervention was principally concerned with family diet and exercise based on existing interventions applied within school settings (BDA, 2003) and was delivered by one of eight therapists (under supervision of an experienced clinical psychologist). MCI/HLC was delivered in parallel with CCBT. As shown in Appendix S3, MCI and HLC were clearly distinct.

### Analysis

A comprehensive statistical analysis plan was prepared before embarking on the analysis (Appendix S4).

In line with our hypotheses and power analysis, we set out to make two sets of pairwise comparisons (MCBT+CCBT vs. CCBT+Con; and MCI+CCBT vs. CCBT+Con). Dichotomous outcome variables (e.g. diagnostic outcomes) were analysed using a modified Poisson regression approach with robust error variance adjusting for the minimisation factors [child age, child gender, type of child anxiety disorder (GAD, social phobia, SAD, other), baseline severity of the child's and the mother's primary anxiety disorder (ADIS Clinician Severity Rating)]. This approach allows for adjusted relative risks to be presented (Zou, 2004). All analyses are presented based on the intention‐to‐treat (ITT) population. Sensitivity analyses of the primary endpoints included: (a) no adjustment for minimisation criteria, (b) per‐protocol population (i.e. those participants who had received at least half of the treatment sessions and had data for the post‐treatment assessments) and (c) multiple imputation analysis (see Appendix S2). Primary outcome results did not change based on the prespecified sensitivity analyses, nor when best‐ and worst‐case scenarios were used in the context of the multiple imputation analysis. Continuous outcomes were modelled using linear regression, adjusted for baseline scores and minimisation factors. No adjustments were made to the confidence intervals or *p*‐values to account for multiplicity.

Current best‐practice methods were adhered to for conducting and reporting economic evaluations alongside trials (Drummond, Sculpher, Claxton, Stoddart, & Torrance, 2015; Husereau et al., 2013; NICE, 2013; Petrou & Gray, 2011). A broad societal perspective cost‐utility analysis framework was adopted for the base case to assess the cost‐effectiveness of (a) CCBT+MCBT compared to CCBT; and (b) CCBT+MCI compared with CCBT. Costs of the nonspecific treatments (NDC/HLC) were excluded in the base‐case analyses. This was in recognition of the fact that the main aim of any economic evaluation is to estimate the likely cost‐effectiveness of a change in clinical practice in real‐world settings, rather than in ideal controlled conditions (as in efficacy trials). Those costs were instead included in some of the sensitivity analyses. Costs were expressed in pounds sterling (£) at 2011/2012 prices. Given the short time‐frame of the trial and follow‐up, discounting was not applied to costs or effects. The base‐case analyses were performed on an ITT basis. Mean imputation methods were used for missing resource use and health outcomes deemed highly deterministic (e.g. face‐to‐face therapists contact), and multiple imputation for other resources (e.g. use of medications), under the assumption of missing at random (Faria, Gomes, Epstein, & White, 2014). For each mother and child participant, all components of treatment costs, stratified by category of resource use and other broader societal costs (educational services, travel costs, time off school/work) were calculated by multiplying units of resource use by their unit costs (see Table S2). These values were then summed to obtain a total cost for each child/mother. Effects were identified and measured using child/mother QALYs, derived from the EQ‐5D‐Y and EQ‐5D‐3L child and mother report, respectively. Incremental mean costs and effects, and the associated 95% CIs, were estimated comparing the two intervention groups for each of the two comparisons (CCBT+MCBT vs. CCBT; and CCBT+MCI vs. CCBT). Incremental cost‐effectiveness ratios (ICERs) were estimated and reported where relevant. Uncertainty in the cost‐effectiveness results was analysed using cost‐effectiveness acceptability curves (CEACs) over a range of potential threshold values that the health system and the wider society might be willing to pay for an additional QALY gained (Fenwick, Marshall, Levy, & Nichol, 2006). Extensive sensitivity analyses were performed for each of the two base‐case comparisons (CCBT+MCBT vs. CCBT; and CCBT+MCI vs. CCBT) and are detailed in Table S3. All analyses were conducted using Stata Software: Release 12, StataCorp 2011.

## Results

Baseline characteristics were well balanced across treatment arms (see Table 2). Numbers of available participants for each treatment arm are shown in Figure 1. No important harms due to any of the interventions or trial procedures were reported during the trial.

**Table 2:** Baseline characteristics by treatment arm

	CCBT+Con *N* = 71	CCBT+MCBT *N* = 69	CCBT+MCI *N* = 71
Child age (mean, *SD*)	10.28 (1.49)	10.29 (1.56)	10.10 (1.68)
Ethnicity *n* (%) White British	67 (94.37)	58 (84.06)	55 (77.46)
Sex *n* (%) Male	34 (47.89)	35 (50.72)	32 (45.07)
Parent marital status *n* (%) married/living with partner	39 (54.93)	50 (72.46)	46 (64.79)
Family socioeconomic status *n* (%) ‘higher’/‘professional’	29 (40.85)	39 (56.52)	38 (53.52)
Child: ADIS‐c/p primary anxiety disorder			
Separation anxiety disorder *n* (%)	19 (26.76)	16 (23.19)	21 (29.58)
Social anxiety disorder *n* (%)	16 (22.54)	18 (26.09)	14 (19.72)
Generalised anxiety disorder *n* (%)	22 (31.00)	20 (28.96)	24 (33.80)
Specific phobia^a^ *n* (%)	8 (11.27)	11 (15.94)	5 (7.04)
Panic disorder ± Agoraphobia *n* (%)	4 (5.63)	2 (2.90)	4 (5.63)
Selective mutism^a^ *n* (%)	0	0	1 (1.41)
Anxiety disorder not otherwise specified *n* (%)	2 (2.82)	2 (2.90)	2 (2.82)
ADIS‐c/p primary diagnosis severity (CSR) Mean (*SD*)	5.65 (0.80)	5.71 (0.79)	5.69 (0.79)
Mother: ADIS primary disorder			
Social anxiety disorder *n* (%)	9 (12.70)	14 (20.3)	11 (15.49)
Generalised anxiety disorder *n* (%)	37 (52.10)	35 (50.7)	40 (56.34)
Specific phobia^a^ *n* (%)	12 (16.90)	17 (24.64)	9 (12.68)
Panic disorder ± Agoraphobia *n* (%)	3 (4.2)	1 (1.4)	3 (4.23)
Other (ADNOS, hypochondriasis, PTSD, OCD)	5 (7.04)	2 (2.9)	8 (11.27)
MDD^b^ *n* (%)	5 (7)	0	0
ADIS primary diagnosis severity (CSR) Mean (*SD*)	5.24 (1.01)	5.17 (0.89)	5.31 (0.98)

ADNOS, anxiety disorder not otherwise specified; CCBT+Con, child cognitive behaviour therapy + nonspecific control interventions; CSR, clinical severity rating; MCBT, CCBT + maternal cognitive behaviour therapy; MCI, CCBT + mother–child interaction treatment; MDD, major depressive disorder; OCD, obsessive‐compulsive disorder; PTSD, post‐traumatic stress disorder.

a Children with specific phobia and selective mutism were only included if also met diagnostic criteria for another comorbid anxiety disorder.

b These mothers had a current anxiety disorder but MDD had the highest severity.

### Manipulation checks

We first examined the extent to which delivery of the adjunct treatments was associated with change in the putative maintenance mechanisms that were targeted; maternal anxiety disorder status and change in maternal cognitions and behaviours. MCBT was successful in changing maternal anxiety disorder status. At Assessment 1B (i.e. after completing MCBT), 58.5% (*n *=* *38) of mothers in the MCBT arm had recovered from their primary diagnosis compared to 36.5% (*n *=* *23) in the CCBT+Con arm (who received NDC) (*adj RR*: 1.63 (95% CI: 1.13, 2.36), *p *=* *.009). The recovery rate in the MCI arm was not different from that in the CCBT+Con arm (who both received NDC) (*adj RR*: 1.22 (95% CI: 0.83, 1.81), *p *=* *.31).

At assessment 2 (i.e. after the children had received CCBT), many of the mothers in the CCBT+Con arm had recovered from their primary disorder and there was, therefore, no longer a superiority in this respect for the CCBT+MCBT arm (CCBT+MCBT‐ CCBT+Con *adj RR*: 1.23 (95% CI: 0.90–1.68, *p *=* *.21; CCBT+MCI‐ CCBT+Con *adj RR*: 1.27 (95% CI: 0.93–1.74, *p *=* *.13).

Change in observed overprotection (across all three mother–child tasks) at the end of all treatment (Assessment 2) was greater in the CCBT+MCI arm compared to CCBT+Con (adjusted mean difference in change from baseline, (−0.03 (−0.06, −0.004), *p *=* *.03). There was no difference in change in observed overprotection between the CCBT+MCBT and CCBT+Con arms (−0.02 (−0.05, 0.009), *p *=* *.17). There were no significant differences between CCBT+Con and either CCBT+MCBT or CCBT+MCI in terms of change in observed intrusiveness, positive behaviours or maternal expressed anxiety (See Table S4 and S5).

Differences between the CCBT+Con and CCBT+MCI arms were also found on change in measures of maternal expectations of how scared the child would be (−0.67 (−1.26, −0.07); *p *=* *.03) and how in control the child would feel (0.53 (0.01, 1.05); *p *=* *.05), with mothers in the CCBT+MCI arm predicting that their child would be less scared and more in control. While a similar pattern was found, the corresponding differences between the CCBT+Con and CCBT+MCBT arms did not quite reach the conventional level of statistical significance (Scared: −0.53 (−1.12, 0.07), *p *=* *.08; Control: 0.50 (−0.03, 1.02), *p *=* *.06). A summary of the results of statistical analyses for maternal anxiety, behaviours and cognitions is given in Table S5.

### Child outcomes

#### Recovery from anxiety diagnoses

Rates of recovery from the primary diagnosis and all anxiety diagnoses are shown in Table 3, and statistical analyses are summarised in Table 4. There were no significant differences between either the CCBT+Con and the CCBT+MCBT (*adj RR*: 1.22 (95% CI: 0.88, 1.67), *p *=* *.23) or the CCBT+MCI arm (*adj RR*: 1.21 (95% CI: 0.88, 1.65), *p = *.24) on the primary outcome, that is recovery from primary anxiety disorder at the post‐treatment assessment (Assessment 2). Similarly, there were no significant post‐treatment differences on recovery from all anxiety disorders between the CCBT+Con and the CCBT+MCBT (1.06 (95% CI: 0.63, 1.78), *p *=* *.82) or CCBT+MCI arms (*adj RR*: 1.48 (95% CI: 0.92, 2.37). At six months post‐treatment (Assessment 3), again there was no difference between the CCBT+Con arm and either the CCBT+MCBT (Free of primary *adj RR* 1.09 (95% CI: 0.81, 1.46), *p *=* *.57; Free of all *adj RR* 1.04 (95% CI: 0.70, 1.53), *p *=* *.86) or the CCBT+MCI arm [Free of primary *adj RR*: 1.26 (95% CI: 0.97, 1.64), *p *=* *.08; Free of all *adj RR*: 1.04 (95% CI: 0.71, 1.55)]. Similarly, at 12 months post‐treatment, there was no difference between the CCBT+Con arm and either the CCBT+MCBT (Free of primary *adj RR*: 0.85 (95% CI: 0.65, 1.12), *p *=* *.26; Free of all *adj RR*: 0.89 (95% CI: 0.61, 1.31), *p *=* *.57) or the CCBT+MCI arm (Free of primary *adj RR*: 1.04 (95% CI: 0.82, 1.30), *p *=* *.77; Free of all *adj RR*: 1.01 (95% CI: 0.70, 1.45), *p *=* *.97).

**Table 3:** Primary and secondary categorical (child) outcomes

	Initial assessment (1)	Post‐treatment assessment (2)	6‐month post‐treatment assessment (3)	12‐month post‐treatment assessment (4)
*n* (%) free of primary diagnosis				
CCBT+ Con	0	27 (48.21)	29 (59.18)	31 (72.09)
CCBT+MCBT	0	35 (58.33)	34 (64.15)	30 (60.00)
CCBT+MCI	0	37 (59.68)	38 (74.51)	34 (73.91)
*n* (%) free of all anxiety diagnoses				
CCBT_+Con	0	16 (28.57)	23 (46.94)	23 (53.49)
CCBT+MCBT	0	18 (30.00)	25 (47.17)	23 (46.00)
CCBT+MCI	0	25 (40.32)	24 (47.06)	24 (52.17)
*n* (%) CGI‐I ‘much’/’very much’ improved				
CCBT +Con	0	36 (64.29)	39 (79.59)	33 (76.74)
CCBT+MCBT	0	48 (80.00)	41 (77.36)	39 (78.00)
CCBT+MCI	0	47 (75.81)	45 (88.24)	37 (80.43)

See Table S1 for descriptive statistics for secondary continuous outcomes.

CCBT+Con, child cognitive behaviour therapy + nonspecific control interventions; CCBT+MCBT, CCBT + maternal cognitive behaviour therapy; CCBT+MCI, CCBT + mother–child interaction treatment; CGI‐I Clinical Global Impression – Improvement.

**Table 4:** Statistical analyses for child primary and secondary categorical outcomes

Parameter		Adjusted RR	95% CI	*p*‐value
Free from primary diagnosis at post‐treatment assessment (2)				
Treatment	CCBT+Con	Ref.		
	CCBT+MCBT	1.18	0.83–1.62	.285
	CCBT+MCI	1.22	0.90–1.67	.203
Free from primary diagnosis at 6‐month post‐treatment assessment (3)				
Treatment	CCBT+Con	Ref.		
	CCBT+MCBT	1.09	0.81–1.46	.566
	CCBT+MCI	1.26	0.97–1.64	.077
Free from primary diagnosis at 12‐month post‐treatment assessment (4)				
Treatment	CCBT+Con	Ref.		
	CCBT+MCBT	0.85	0.65–1.12	.257
	CCBT+MCI	1.04	0.82–1.30	.766
Free from all anxiety diagnoses at post‐treatment assessment (2)				
Treatment	CCBT+Con	Ref.		
	CCBT+MCBT	1.06	0.63–1.78	.816
	CCBT+MCI	1.48	0.92–2.37	.102
Free from all anxiety diagnoses at 6‐month post‐treatment assessment (3)				
Treatment	CCBT+Con	Ref.		
	CCBT+MCBT	1.04	0.70–1.53	.860
	CCBT+MCI	1.04	0.71–1.55	.814
Free from all anxiety diagnoses at 12‐month post‐treatment assessment (4)				
Treatment	CCBT+Con	Ref.		
	CCBT+MCBT	0.89	0.61–1.31	.569
	CCBT+MCI	1.01	0.70–1.45	.972
CGI‐I ‘much’/’very much’ improved at post‐treatment assessment (2)				
Treatment	CCBT+Con	Ref.		
	CCBT+MCBT	1.26	1.00–1.59	.054
	CCBT+MCI	1.20	0.95–1.53	.133
CGI‐I ‘much’/’very much’ improved at 6‐month post‐treatment assessment (3)				
Treatment	CCBT+Con	Ref.		
	CCBT+MCBT	0.97	0.79–1.19	.771
	CCBT+MCI	1.16	0.94–1.33	.216
CGI‐I ‘much’/’very much’ improved at 12‐month post‐treatment assessment (4)				
Treatment	CCBT+Con	Ref.		
	CCBT+MCBT	1.02	0.82–1.27	.834
	CCBT+MCI	1.05	0.85–1.30	.628

Adjusted for child age, child gender, type of child anxiety disorder (GAD, social phobia, SAD, other), baseline severity (ADIS‐C/P CSR) of the child's primary anxiety disorder and baseline severity (ADIS‐IV mother self‐report) of the mother's primary anxiety disorder.

CCBT+Con, child cognitive behaviour therapy + nonspecific control interventions; CCBT+MCBT, CCBT + maternal cognitive behaviour therapy; CCBT+MCI, CCBT + mother–child interaction treatment; CGI‐I, Clinical Global Impression – Improvement; Ref., reference category.

**Table 5:** Statistical analyses for child secondary continuous outcomes

	Treatment arm	*N*	Adjusted^a^ Mean change (95% CI)	Adjusted^a^ Mean difference (95% CI)	*p* value
Anxiety symptoms (SCAS‐c) – mid‐treatment assessment (1B)	CCBT+Con	53	−7.71 (−10.87, −4.56)	Ref	
	CCBT+MCBT	60	−6.15 (−9.13, −3.17)	1.57 (−2.82, 5.96)	.482
	CCBT+MCI	64	−6.02 (−8.89, −3.15)	1.69 (−2.61, 5.99)	.438
Anxiety symptoms (SCAS‐c) – post‐treatment assessment (2)	CCBT+Con	45	−19.68 (−23.48, −15.89)	Ref	
	CCBT+MCBT	46	−13.71 (−17.49, −9.92)	5.97 (0.54, 11.41)	.031
	CCBT+MCI	52	−15.73 (−19.27, −12.19)	3.96 (−1.28, 9.19)	.137
Anxiety symptoms (SCAS‐c) – 6‐month post‐treatment assessment (3)	CCBT+Con	41	−17.85 (−22.73, −12.98)	Ref	
	CCBT+MCBT	43	−16.59 (−21.35, −11.83)	1.26 (−5.56, 8.08)	.715
	CCBT+MCI	41	−17.60 (−22.52, −12.67)	0.26 (−6.77, 7.28)	.943
Anxiety symptoms (SCAS‐c) – 12‐month post‐treatment assessment (4)	CCBT+Con	31	−18.11 (−23.18, −13.03)	Ref	
	CCBT+MCBT	34	−18.92 (−23.82, −14.01)	−0.81 (−7.96, 6.34)	.823
	CCBT+MCI	37	−17.98 (−22.59, −13.38)	0.12 (−6.73, 6.97)	.972
Anxiety symptoms (SCAS‐p) – mid‐treatment assessment (1B)	CCBT+Con	42	−8.30 (−11.68, −4.93)	Ref	
	CCBT+MCBT	44	−8.14 (−11.45, −4.83)	0.17 (−4.62, 4.96)	.944
	CCBT+MCI	48	−9.38 (−12.54, −6.21)	−1.07 (−5.75, 3.62)	.653
Anxiety symptoms (SCAS‐p) – post‐treatment assessment (2)	CCBT+Con	36	−18.00 (−21.09, −14.88)	Ref	
	CCBT+MCBT	39	−16.77 (−19.75, −13.78)	1.22 (−3.15, 5.59)	.581
	CCBT+MCI	38	−18.30 (−21.35, −15.25)	−0.32 (−4.77, 4.14)	.888
Anxiety symptoms (SCAS‐p) – 6‐month post‐treatment assessment (3)	CCBT+Con	36	−17.44 (−21.03, −13.84)	Ref	
	CCBT+MCBT	41	−16.62 (−20.00, −13.25)	0.82 (−4.11, 5.74)	.743
	CCBT+MCI	38	−19.17 (−22.72, −15.61)	−1.73 (−6.86, 3.40)	.505
Anxiety symptoms (SCAS‐p) – 12‐month post‐treatment assessment (4)	CCBT+Con	30	−22.37 (−26.62, −18.12)	Ref	
	CCBT+MCBT	31	−16.36 (−20.58, −12.15)	6.01 (−0.01, 12.02)	.050
	CCBT+MCI	33	−20.74 (−24.78, −16.71)	1.62 (−4.27, 7.51)	.585
Anxiety symptoms (SCAS‐t) – post‐treatment assessment (2)	CCBT+Con	7	−4.02 (−11.27, 3.23)	Ref	
	CCBT+MCBT	14	−4.94 (−9.90, 0.03)	−0.92 (−10.69, 8.85)	.847
	CCBT+MCI	12	−5.31 (−10.53, −0.10)	−1.29 (−10.86, 8.27)	.782
Anxiety symptoms (SCAS‐t) – 6‐month post‐treatment assessment (3)	CCBT+Con	4	−1.88 (−15.75, 12.00)	Ref	
	CCBT+MCBT	9	−15.07 (−22.85, −7.29)	−13.19 (−30.33, 3.94)	.123
	CCBT+MCI	15	−10.26 (−15.89, −4.64)	−8.39 (−24.79, 8.01)	.296
Anxiety symptoms (SCAS‐t) – 12‐month post‐treatment assessment (4)	CCBT+Con	4	−7.90 (−17.17, 1.37)	Ref	
	CCBT+MCBT	4	−0.04 (−18.30, 18.22)	7.86 (−12.83, 28.55)	.244
	CCBT+MCI	5	−11.90 (−23.14, −0.66)	−4.00 (−18.25, 10.25)	.351
Child Anxiety Impact Scale (CAIS‐c) – mid‐treatment assessment (1B)	CCBT+Con	52	−4.30 (−7.36, −1.24)	Ref	
	CCBT+MCBT	58	−6.48 (−9.41, −3.55)	−2.18 (−6.51, 2.15)	.322
	CCBT+MCI	62	−7.19 (−9.97, −4.41)	−2.89 (−7.03, 1.26)	.171
Child Anxiety Impact Scale (CAIS‐c) – post‐treatment assessment (2)	CCBT+Con	44	−8.19 (−12.10, −4.28)	Ref	
	CCBT+MCBT	45	−6.28 (−10.20, −2.37)	1.91 (−3.73, 7.55)	.505
	CCBT+MCI	53	−6.49 (−10.05, −2.93)	1.70 (−3.63, 7.03)	.530
Child Anxiety Impact Scale (CAIS‐c) – 6‐month post‐treatment assessment (3)	CCBT+Con	38	−12.45 (−15.06, −9.84)	Ref	
	CCBT+MCBT	43	−11.02 (−13.50, −8.54)	1.43 (−2.21, 5.07)	.437
	CCBT+MCI	41	−10.75 (−13.29, −8.21)	1.70 (−1.97, 5.37)	.361
Child Anxiety Impact Scale (CAIS‐c) – 12‐month post‐treatment assessment (4)	CCBT+Con	29	−12.83 (−17.14, −8.51)	Ref	
	CCBT+MCBT	33	−9.25 (−13.41, −5.09)	3.57 (−2.59, 9.74)	.253
	CCBT+MCI	37	−11.71 (−15.48, −7.93)	1.12 (−4.59, 6.83)	.698
Child Anxiety Impact Scale (CAIS‐p) – mid‐treatment assessment (1B)	CCBT+Con	39	−2.39 (−4.69, −0.08)	Ref	
	CCBT+MCBT	39	−5.57 (−7.89, −3.25)	−3.18 (−6.51, 0.15)	.061
	CCBT+MCI	40	−4.77 (−7.05, −2.48)	−2.38 (−5.67, 0.91)	.154
Child Anxiety Impact Scale (CAIS‐p) – post‐treatment assessment (2)	CCBT+Con	33	−10.19 (−12.37, −8.01)	Ref	
	CCBT+MCBT	35	−12.95 (−15.09, −10.80)	−2.76 (−5.86, 0.34)	.080
	CCBT+MCI	31	−10.15 (−12.45, −7.84)	0.04 (−3.20, 3.28)	.980
Child Anxiety Impact Scale (CAIS‐p) – 6‐month post‐treatment assessment (3)	CCBT+Con	35	−9.25 (−12.12, −6.37)	Ref	
	CCBT+MCBT	37	−12.13 (−14.95, −9.31)	−2.88 (−6.92, 1.16)	.160
	CCBT+MCI	34	−9.69 (−12.68, −6.70)	−0.44 (−4.61, 3.73)	.835
Child Anxiety Impact Scale (CAIS‐p) – 12‐month post‐treatment assessment (4)	CCBT+Con	28	−9.50 (−12.44, −6.56)	Ref	
	CCBT+MCBT	27	−12.11 (−15.19, −9.04)	−2.61 (−6.88, 1.66)	.227
	CCBT+MCI	31	−12.15 (−14.99, −9.32)	−2.65 (−6.72, 1.42)	.199
Depression symptoms (SMFQ‐c) – mid‐treatment assessment (1B)	CCBT+Con	52	−2.83 (−3.96, −1.70)	Ref	
	CCBT+MCBT	60	−2.09 (−3.15, −1.03)	0.74 (−0.82, 2.30)	.350
	CCBT+MCI	64	−2.48 (−3.50, −1.45)	0.36 (−1.18, 1.90)	.649
Depression symptoms (SMFQ‐c) – post‐treatment assessment (2)	CCBT+Con	46	−5.03 (−6.35, −3.71)	Ref	
	CCBT+MCBT	47	−2.25 (−3.57, −0.93)	2.78 (0.88, 4.68)	.004
	CCBT+MCI	54	−2.70 (−3.92, −1.48)	2.33 (0.52, 4.14)	.012
Depression symptoms (SMFQ‐c) – 6‐month post‐treatment assessment (3)	CCBT+Con	40	−3.81 (−5.33, −2.29)	Ref	
	CCBT+MCBT	44	−3.52 (−4.97, −2.06)	0.30 (−1.82, 2.42)	.783
	CCBT+MCI	39	−3.97 (−5.52, −2.41)	−0.16 (−2.36, 2.04)	.887
Depression symptoms (SMFQ‐c) – 12‐month post‐treatment assessment (4)	CCBT+Con	28	−4.08 (−5.95, −2.21)	Ref	
	CCBT+MCBT	35	−4.20 (−5.91, −2.49)	−0.12 (−2.71, 2.48)	.928
	CCBT+MCI	37	−2.09 (−3.72, −0.47)	1.99 (−0.48, 4.46)	.113
Depression symptoms (SMFQ‐p) – mid‐treatment assessment (1B)	CCBT+Con	40	−1.32 (−2.97, 0.32)	Ref	
	CCBT+MCBT	38	−3.17 (−4.86, −1.47)	−1.85 (−4.24, 0.55)	.130
	CCBT+MCI	44	−0.63 (−2.20, 0.94)	0.69 (−1.61, 3.00)	.552
Depression symptoms (SMFQ‐p) – post‐treatment assessment (2)	CCBT+Con	34	−4.60 (−6.03, −3.18)	Ref	
	CCBT+MCBT	38	−5.66 (−7.03, −4.29)	−1.06 (−3.07, 0.96)	.301
	CCBT+MCI	35	−5.64 (−7.07, −4.22)	−1.04 (−3.08, 1.00)	.314
Depression symptoms (SMFQ‐p) – 6‐month post‐treatment assessment (3)	CCBT+Con	36	−4.25 (−5.82, −2.67)	Ref	
	CCBT+MCBT	38	−5.86 (−7.40, −4.32)	−1.61 (−3.83, 0.61)	.154
	CCBT+MCI	36	−4.90 (−6.49, −3.30)	−0.65 (−2.91, 1.61)	.570
Depression symptoms (SMFQ‐p) – 12‐month post‐treatment assessment (4)	CCBT+Con	30	−5.14 (−6.81, −3.48)	Ref	
	CCBT+MCBT	29	−4.54 (−6.25, −2.82)	0.61 (−1.82, 3.04)	.619
	CCBT+MCI	33	−5.97 (−7.55, −4.39)	−0.83 (−3.11, 1.46)	.474
Conduct problems (SDQ‐c) – mid‐treatment assessment (1B)	CCBT+Con	54	−0.48 (−0.88, −0.08)	Ref	
	CCBT+MCBT	58	−0.18 (−0.56, 0.21)	0.30 (−0.26, 0.86)	.289
	CCBT+MCI	65	−0.26 (−0.62, 0.11)	0.22 (−0.33, 0.77)	.427
Conduct problems (SDQ‐c) – post‐treatment assessment (2)	CCBT+Con	47	−0.61 (−1.08, −0.14)	Ref	
	CCBT+MCBT	47	−0.52 (−1.00, −0.05)	0.09 (−0.59, 0.76)	.803
	CCBT+MCI	55	−0.50 (−0.94, −0.07)	0.10 (−0.54, 0.75)	.748
Conduct problems (SDQ‐c) – 6‐month post‐treatment assessment (3)	CCBT+Con	42	−0.88 (−1.35, −0.42)	Ref	
	CCBT+MCBT	44	−0.91 (−1.37, −0.46)	−0.03 (−0.68, 0.62)	.925
	CCBT+MCI	42	−0.81 (−1.28, −0.34)	0.07 (−0.60, 0.74)	.832
Conduct problems (SDQ‐c) – 12‐month post‐treatment assessment (4)	CCBT+Con	31	−1.21 (−1.87, −0.55)	Ref	
	CCBT+MCBT	34	−0.94 (−1.58, −0.30)	0.27 (−0.65, 1.18)	.562
	CCBT+MCI	38	−1.00 (−1.59, −0.40)	0.21 (−0.68, 1.10)	.637
Conduct problems (SDQ‐p) – mid‐treatment assessment (1B)	CCBT+Con	42	−0.14 (−0.54, 0.26)	Ref	
	CCBT+MCBT	46	−0.18 (−0.56, 0.21)	−0.04 (−0.60, 0.52)	.897
	CCBT+MCI	50	−0.08 (−0.45, 0.29)	0.06 (−0.49, 0.61)	.836
Conduct problems (SDQ‐p) – post‐treatment assessment (2)	CCBT+Con	37	−0.65 (−1.06, −0.24)	Ref	
	CCBT+MCBT	41	−0.74 (−1.12, −0.35)	−0.09 (−0.66, 0.49)	.763
	CCBT+MCI	40	−0.84 (−1.23, −0.45)	−0.19 (−0.77, 0.39)	.515
Conduct problems (SDQ‐p) – 6‐month post‐treatment assessment (3)	CCBT+Con	39	−0.47 (−0.86, −0.09)	Ref	
	CCBT+MCBT	42	−0.99 (−1.36, −0.62)	−0.51 (−1.05, 0.02)	.060
	CCBT+MCI	41	−1.11 (−1.49, −0.73)	−0.64 (−1.19, −0.09)	.022
Conduct problems (SDQ‐p) – 12‐month post‐treatment assessment (4)	CCBT+Con	32	−1.04 (−1.56, −0.53)	Ref	
	CCBT+MCBT	32	−0.89 (−1.40, −0.38)	0.16 (−0.57, 0.89)	.668
	CCBT+MCI	38	−0.85 (−1.31, −0.38)	0.20 (−0.50, 0.89)	.575
Conduct problems (SDQ‐t) – post‐treatment assessment (2)	CCBT+Con	18	0.35 (−0.21, 0.91)	Ref	
	CCBT+MCBT	22	−0.17 (−0.68, 0.35)	−0.52 (−1.30, 0.27)	.190
	CCBT+MCI	23	−0.11 (−0.62, 0.39)	−0.46 (−1.24, 0.31)	.236
Conduct problems (SDQ‐t) – 6‐month post‐treatment assessment (3)	CCBT+Con	12	0.57 (−0.36, 1.50)	Ref	
	CCBT+MCBT	18	−0.21 (−0.98, 0.57)	−0.78 (−1.98, 0.42)	.196
	CCBT+MCI	22	0.31 (−0.37, 1.00)	−0.26 (−1.42, 0.90)	.654
Conduct problems (SDQ‐t) – 12‐month post‐treatment assessment (4)	CCBT+Con	9	−0.09 (−1.21, 1.03)	Ref	
	CCBT+MCBT	11	−0.27 (−1.30, 0.75)	−0.18 (−1.71, 1.34)	.805
	CCBT+MCI	12	0.41 (−0.54, 1.36)	0.50 (−0.99, 2.00)	.490
Child Adjustment to School (CAS‐t) – post‐treatment assessment (2)	CCBT+Con	18	−0.86 (−2.32, 0.60)	Ref	
	CCBT+MCBT	24	−1.96 (−3.22, −0.70)	−1.10 (−3.11, 0.90)	.275
	CCBT+MCI	25	−1.26 (−2.48, −0.04)	−0.40 (−2.37, 1.57)	.684
Child Adjustment to School (CAS‐t) – 6‐month post‐treatment assessment (3)	CCBT+Con	11	−1.43 (−3.85, 0.99)	Ref	
	CCBT+MCBT	17	−2.92 (−4.91, −0.94)	−1.50 (−4.65, 1.66)	.343
	CCBT+MCI	23	−1.40 (−3.02, 0.23)	0.03 (−2.92, 2.98)	.985
Child Adjustment to School (CAS‐t) – 12‐month post‐treatment assessment (4)	CCBT+Con	9	−2.56 (−4.76, −0.37)	Ref	
	CCBT+MCBT	10	−0.04 (−2.23, 2.14)	2.52 (−0.62, 5.66)	.110
	CCBT+MCI	12	−0.40 (−2.29, 1.48)	2.16 (−0.77, 5.09)	.139

CAIS‐c/p, Child Anxiety Impact Scale child/parent report; CCBT+Con, child cognitive behaviour therapy + nonspecific control interventions; CCBT+MCBT, CCBT + maternal cognitive behaviour therapy; CCBT+MCI, CCBT + mother–child interaction treatment; SCAS‐c/p, Spence Children's Anxiety Scale – child/parent report; SDQ‐p, Strengths and Difficulties Questionnaire – parent report; SMFQ‐c/p, Short Mood and Feelings Questionnaire – child/parent report.

a Adjusted for child age, child gender, type of child anxiety disorder (GAD, social phobia, SAD, other), baseline severity (ADIS Clinician Severity Rating) of the child's primary anxiety disorder, baseline severity (ADIS Mother self‐report) of the mother's primary anxiety disorder and baseline questionnaire score.

#### Clinical Global Impressions of Improvement (CGI‐I)

As shown in Table 4, at the post‐treatment assessment (Assessment 2), there were no significant differences between the groups in terms of rates of ‘much’ or ‘very much’ improvement (CCBT+MCBT vs. CCBT+Con *adj RR*: 1.25 (95% CI: 0.99, 1.57), *p *=* *.06; CCBT+MCI vs. CCBT+Con *adj RR*: 1.18 (95% CI: 0.93, 1.50), *p *=* *.17). Differences were even smaller at the six month (Assessment 3) (CCBT+MCBT vs. CCBT+Con *adj* RR: 0.97 (95% CI: 0.79, 1.19), *p *=* *.77; CCBT+MCI vs. CCBT+Con *adj RR*: 1.16 (95% CI: 0.94, 1.33), *p *=* *.22) and 12 months post‐treatment assessment (Assessment 4; CCBT+MCBT vs. CCBT+Con *adj RR*: 1.02 (95% CI: 0.82, 1.27), *p* = .83; CCBT+Con vs. CCBT+MCI *adj RR*: 1.05 (95% CI: 0.85, 1.30), *p *=* *.63).

### Economic evaluation

Missing resource use data were prevalent (Table S6). Mother and child combined HRQoL results are presented in Table S7 (unadjusted differences) and Tables S8 and S9 (differences adjusted for child's and mother's baseline utilities). At 12 months, in the base‐case analysis, the CCBT+Con arm conferred slightly higher QALYs than the CCBT+MCBT arm (CCBT+MCBT vs. CCBT+Con: −0.04 (95% CI: −0.12, 0.04), *p *=* *.29), whilst the CCBT+MCI arm conferred slightly higher QALYs than the CCBT+Con arm (CCBT+MCI vs. CCBT+Con: 0.02 (95% CI: −0.05, 0.09), *p *=* *.54). Neither of these differences was statistically significant. Mean NHS and societal cost differences for the two comparisons are reported in Tables S10–S12. When a societal perspective was adopted (base‐case analysis; Table S11), CCBT+MCBT was £482 more costly than CCBT (95% CI: −£827, £1,791); this was not significant (*p* = .47). However, from an NHS perspective, costs for CCBT+MCBT were £797 significantly greater than CCBT (95% CI: £603, £991; *p *<* *.001). For CCBT+MCI, when a societal perspective was adopted [base‐case analysis; Table S10), CCBT+MCI was £154 more costly than CCBT (95% CI: −£1,239, £1,547)], again an insignificant difference (*p* = .83). The NHS perspective costs for CCBT+MCI, on the other hand, were £808 greater than for CCBT (95% CI: £610, £1,006), a statistically significant difference (*p *<* *.001).

Key cost drivers in both of these base‐case comparisons were the differential treatment delivery costs in both the CCBT+MBCT and CCBT+MCI arms compared with CCBT only (Tables S11 and S12). While the CCBT+Con arm had higher absences from school and work costs than the other two arms, few differences were statistically significant although, notably, the lower costs of mother's lost days of work in the CCBT+MCI arm than in the CCBT+Con arm were significant (Table S12).

**Figure 2:**
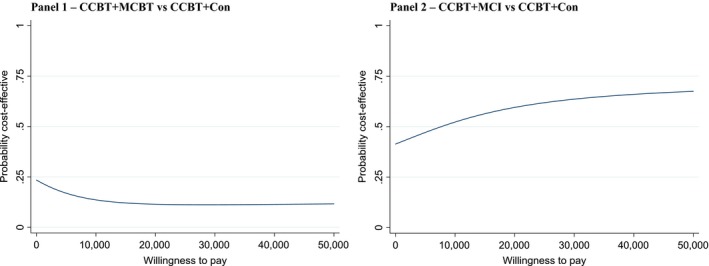
Cost‐effectiveness acceptability curves for the base‐case analyses. CCBT+Con, child‐focused cognitive behaviour therapy + nonspecific control interventions; CCBT+ MCBT, CCBT + CBT to target maternal anxiety disorder; CCBT+MCI, CCBT + intervention to target the mother–child interaction [Colour figure can be viewed at http://www.wileyonlinelibrary.com]

Taking sampling uncertainty into consideration, the cost‐effectiveness acceptability curves (CEACs) for the two base‐case analyses showed that, in view of the joint distribution of incremental mean costs and effects, the CCBT+MBCT arm was not likely to be a cost‐effective alternative to CCBT (Figure 2, Panel 1, and Table S8, base‐case line). The probability that the CCBT+MCI arm was cost‐effective compared to CCBT (Figure 2, Panel 2, and Table S9 base‐case line) was between 60% and 64% based on UK National Institute for Health and Care Excellence thresholds for accepted levels of willingness to pay for an additional QALY (usually between £20,000 and £30,000). Sensitivity analyses reinforced the findings of the base‐case analyses, with the CCBT+MCBT arm not being cost‐effective compared with the CCBT arm across the majority of sensitivity analyses (Table S8) [other than the scenario where the CCBT+MCBT arm was less costly but also less effective (SA 4 and SA 10)]. For the comparison between the CCBT+MCI arm and CCBT (Table S9), the CCBT+MCI arm may be a cost‐effective alternative to CCBT from a societal perspective (insignificantly higher costs or less costly, insignificantly higher QALYs), as reflected by the probability of cost‐effectiveness being greater than 60% overall.

## Discussion

Good outcomes were achieved for both children and their mothers across all treatment arms but neither adding treatment of maternal anxiety (MCBT) nor treatment of the mother–child relationship (MCI) to individual child‐focused CBT conferred a significant benefit to children with anxiety disorders whose mothers also had a current anxiety disorder. Although adding both adjunct treatments achieved higher child recovery rates post‐treatment than the CCBT plus control treatment arm (where neither maternal anxiety nor potentially anxiogenic parenting received specific therapeutic attention), the advantages were not statistically significant. However, from a broad societal perspective combining mother/child QALYs and costs, including productivity and school impacts, the economic evaluation indicated that CCBT+MCI arm (but not the CCBT+MCBT arm) may be cost‐effective compared to CCBT. Notably, the probability of the CCBT+MCI arm being cost‐effective lessened when a more restricted NHS perspective was adopted and the gains from reduced school absenteeism and mothers’ productivity/employment excluded. This indicates that costs associated with childhood anxiety disorders are likely to be underestimated if a purely healthcare provider perspective is adopted, and it affirms the importance of including wider family/society born costs and outcomes in mental health economic evaluations (Creswell et al., 2017; Tilford et al., 2015; Tubeuf & Guthmuller, 2017).

Despite these potential broader economic benefits, it is important to consider the lack of significant clinical effects of the two active adjunct interventions (MCI/MCBT). The study was powered to detect a 30% difference in the proportion of recovered children, based on a projected recovery rate from child CBT of 40% (Cobham et al., 1998). While a 30% difference in recovery between CBT plus the adjunct treatments and CBT plus the control interventions might be considered conservative (as smaller differences may still be clinically meaningful), an anticipated success rate of 40% from individual CBT could have been considered optimistic, given findings from a more recent trial – the first to examine treatment of child anxiety disorder in the context of diagnosed parental anxiety disorder – where a recovery rate of only 33% was found (Hudson et al., 2014). However, the CCBT plus control treatment arm in the current study performed considerably better than expected, with 48% of children being free of their primary anxiety disorder and 64% ‘much’ or ‘very much’ improved immediately post‐treatment, and over 70% free of their primary diagnosis and ‘much/very much improved’ a year after treatment. Indeed, these success rates are similar to those found from more intensive (14 session) CCBT for children with anxiety disorders, regardless of parental anxiety disorder status, where the proportions ‘much’/’very much’ improved were 60% at post‐treatment (Walkup et al., 2008) and 72% at six‐month follow‐up (Piacentini et al., 2014), respectively. The unexpectedly high rate of recovery within the CCBT plus control arm in the current study is unlikely to be a function of particular features of our sample, as our study population was comparable to other clinic samples in the literature. There could, however, have been an added value of the nonspecific interventions, all of which provided some level of parental support. This raises the possibility that the modest outcomes typically found for anxious children in the context of parental anxiety disorder are in fact a result of other associated factors – for example, other stressors experienced by the family (see, e.g. Schleider et al., 2015) – which might have been addressed to some extent in this trial by the generic support received by all mothers.

Given the novelty of the adjunctive treatments, the degree to which they were successful in altering their respective targets also needs to be considered. In the one previous study that assessed the impact of adding CBT for parental anxiety disorders to CBT for child anxiety disorders (Hudson et al., 2014), the failure to find significant differences in child outcomes may have been attributable to the parental CBT not reducing parental anxiety. In the current study, however, compared to nondirective counselling, MCBT was associated with a significant reduction in the frequency of maternal anxiety disorder. However, after the children had received treatment, there was no longer a significant difference in maternal disorder status, with all treatment arms showing relatively high rates of recovery from maternal disorders. This is consistent with previous findings indicating that reductions in child anxiety are associated with reductions in parental anxiety (Settipani et al., 2013).

We conducted observational assessments of parental responses before and after treatment for child anxiety disorders which provided evidence that, in terms of a relative change in overprotective behaviours, the mother–child interaction treatment was successful. The MCI intervention was also associated with change in maternal cognitions associated with confidence in child coping (i.e. reduced predictions regarding child fear and increased predictions regarding child control). Despite these positive benefits, no significant benefit to child outcome was conferred. Possible reasons for this may be that the changes were not of sufficient magnitude to be of benefit, or, as noted above, that change in these factors is not in fact critical to changing child anxiety. Notably, there was no specific benefit for the MCI intervention on measures of maternal expressed anxiety, intrusiveness or positive behaviours. While it is possible that the intervention was ineffective with respect to these dimensions, it may well be that these parental behaviours changed equally across groups in *response* to improvements in child anxiety (Silverman, Kurtines, Jaccard, & Pina, 2009). However, it may also reflect limitations in the extent to which these laboratory‐based observational tasks assess parenting dimensions as they are expressed in the home.

The study had several notable strengths, including the use of reliable, blind raters to make assessments of child and maternal anxiety and maternal behaviours and cognitions before and after treatment, the inclusion of nonspecific interventions to balance therapist contact, a design which allowed for isolating the effects of specifically targeting maternal anxiety and parenting responses, and the inclusion of a broad societal perspective, prospectively designed economic evaluation measuring and valuing both mother and child costs and outcomes. These strengths need to be considered in the light of various limitations. Although we allowed for 20% loss to follow‐up, by the one year post‐treatment, assessment retention was down to 61% in the CCBT+Con arm. Although there were no clear baseline differences between completers and those who dropped out, it is of concern that the greatest drop‐out occurred during the eight‐session maternal counselling phase which may not have been a sufficiently acceptable treatment approach for some families. Whether dropouts overrepresented those with good or bad treatment outcomes cannot be determined, although the sensitivity analyses that were conducted gave a consistent pattern of results which suggest that this was not the case. Other limitations include the relatively restricted demographic characteristics of the participants, who were predominantly of nonminority ethnicity and relatively high socioeconomic status. We elected to focus on middle childhood (7–12 years) as anxiety disorders can be reliably diagnosed at this age, particular parental behaviours have been observed in the context of parent anxiety disorder at this age (Creswell et al., 2013), and it is likely that the nature of parental influences on child anxiety varies with child age (Connell & Goodman, 2002; Maccoby, 1992). We also focused on intervening with mothers as parental influences have also been shown to differ according to parent gender (Bögels & Perotti, 2011). As a result, however, the findings cannot be generalised to younger children, to adolescents or to interventions with fathers or other caregivers. The study also included children and mothers with a broad range of anxiety disorders, and further work needs to be done which takes account of the precise form of parental and child anxiety.

For the health economics analyses, data on resource use and costs of additional health and personal social services beyond the NHS treatment costs were characterised by a high percentage of missing data. However, the economic results were confirmed by multiple sensitivity analyses, including a variation that best approximated a complete‐case scenario. The base‐case economic evaluations excluded the costs of the two nonspecific interventions (i.e. family healthy living and nondirective counselling for mothers), which were introduced to match for therapist time/contact across the trial arms, and are not commonly delivered in ‘real‐world’ settings. While the sensitivity analyses that were conducted including those costs confirmed and reinforced the base‐case results, it should be acknowledged that the nonspecific interventions may not have been completely neutral in their effect. However, in the absence of a valid counterfactual, we are unable to quantify that potential impact. Furthermore, this study should be regarded as providing an indication of the short‐term likely cost‐effectiveness of CCBT+MCBT or CCBT+MCI compared to CCBT, with further research required to determine cost‐effectiveness in the longer term. Finally, generalising these economic findings to other populations and settings (e.g. different healthcare systems) is challenging and, with the added complexity associated with capturing the broader societal impact as well as the combined mother/child impacts, the cost‐effectiveness results are best interpreted with caution.

In sum, the current study showed that good clinical outcomes can be achieved for children with anxiety disorders in the context of maternal anxiety disorder by providing high‐quality individual child CBT together with some parental support. However, although offering CBT to mothers for their own anxiety disorders gave an early boost to the timing of maternal recovery, it did not lead to a significant clinical benefit in terms of child anxiety treatment outcome and (given the higher costs) was not cost‐effective. An adjunctive treatment to target parenting responses had a nonsignificant positive effect on child outcomes, which, despite the higher costs, may be cost‐effective. The findings also suggest that reductions in child anxiety may have a positive effect on maternal mental health.

**Key points**

- Treatment outcomes for children with anxiety disorders are impaired in the context of concurrent parental anxiety disorder but it is unclear how to improve outcomes.
- In this RCT for children with anxiety disorders whose mothers also had a current anxiety disorder, individual child‐focused CBT was supplemented with treatments to (a) improve maternal anxiety (MCBT) or (b) alter maternal responses to her child when faced with challenge (MCI).
- The adjunct treatments were successful in improving maternal anxiety and responses; however, this did not lead to a significant benefit in terms of child anxiety treatment outcome.
- Good child outcomes were achieved for children with anxiety disorders in the context of maternal anxiety disorder by providing high‐quality individual child CBT together with some parental support.
- Reductions in child anxiety may also have a positive effect on maternal mental health.
- MCI (but not MCBT) may be a cost‐effective psychological approach for the treatment of child anxiety problems in the context of maternal anxiety disorders.

## Supporting information


**Appendix S1.** Study protocol.
**Appendix S2.** Statistical analysis plan.
**Appendix S3.** Adherence and fidelity.
**Appendix S4.** Multiple imputation approach.
**Table S1.** Scores at each time point on continuous secondary outcome measures.
**Table S2.** Unit costs.
**Table S3.** Sensitivity analyses for base‐case comparisons.
**Table S4.** Descriptive data: Maternal anxiety disorders, behaviours and cognitions.
**Table S5.** Manipulation checks: Statistical analyses for maternal anxiety disorders, behaviours and cognitions.
**Table S6.** Health Economic data completeness (percentage of missing data reported).
**Table S7.** Child and mother combined Quality Adjusted Life Years (QALYs) gained – CCBT+MCBT versus CCBT+Con; CCBT+MCI versus CCBT+Con.
**Table S8.** Cost utility analysis – CCBT+MCBT versus CCBT(+Con).
**Table S9.** Cost utility analysis – CCBT+MCI versus CCBT(+Con).
**Table S10.** Societal cost mean differences – CCBT+MCBT versus CCBT; CCBT+ MCI versus CCBT(+Con).
**Table S11.** Treatment resource use mean differences – CCBT+MCBT versus CCBT+Con.
**Table S12.** Treatment resource use mean differences – CCBT+MCI versus CCBT+Con.
