# Supplementary material for: A randomised controlled trial of treatments of childhood anxiety disorder in the context of maternal anxiety disorder: clinical and cost‐effectiveness outcomes
Source: J Child Psychol Psychiatry. 2019 Jul 31;61(1):62–76. doi: 10.1111/jcpp.13089 (PMC6916180; doi:10.1111/jcpp.13089)
Supplement: Supplementary file 1 — Appendix S1. Study protocol. Appendix S2. Statistical analysis plan. Appendix S3. Adherence and fidelity. Appendix S4. Multiple imputation approach. Table S1. Scores at each time point on continuous secondary outcome measures. Table S2. Unit costs. Table S3. Sensitivity analyses for base‐case comparisons. Table S4. Descriptive data: Maternal anxiety disorders, behaviours and cognitions. Table S5. Manipulation checks: Statistical analyses for maternal anxiety disorders, behaviours and cognitions. Table S6. Health Economic data completeness (percentage of missing data reported). Table S7. Child and mother combined Quality Adjusted Life Years (QALYs) gained – CCBT+MCBT versus CCBT+Con; CCBT+MCI versus CCBT+Con. Table S8. Cost utility analysis – CCBT+MCBT versus CCBT(+Con). Table S9. Cost utility analysis – CCBT+MCI versus CCBT(+Con). Table S10. Societal cost mean differences – CCBT+MCBT versus CCBT; CCBT+ MCI versus CCBT(+Con). Table S11. Treatment resource use mean differences – CCBT+MCBT versus CCBT+Con. Table S12. Treatment resource use mean differences – CCBT+MCI versus CCBT+Con. [file JCPP-61-62-s001.docx]

**Supporting information – A randomised controlled trial of treatments of childhood anxiety disorder in the context of maternal anxiety disorder: clinical and cost-effectiveness outcomes – by Creswell et al.**


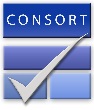
CONSORT 2010 checklist of information to include when reporting a randomised trial*

| Section/Topic | Item No | Checklist item | Reported on page No |
| --- | --- | --- | --- |
| Title and abstract | | | |
|  | 1a | Identification as a randomised trial in the title | 1 |
|  | 1b | Structured summary of trial design, methods, results, and conclusions (for specific guidance see CONSORT for abstracts) | 2-3 |
| Introduction | | | |
| Background and objectives | 2a | Scientific background and explanation of rationale | 4-6 |
|  | 2b | Specific objectives or hypotheses | 5-6 |
| Methods | | | |
| Trial design | 3a | Description of trial design (such as parallel, factorial) including allocation ratio | 8 |
|  | 3b | Important changes to methods after trial commencement (such as eligibility criteria), with reasons | n/a |
| Participants | 4a | Eligibility criteria for participants | 6-7 |
|  | 4b | Settings and locations where the data were collected | 7 |
| Interventions | 5 | The interventions for each group with sufficient details to allow replication, including how and when they were actually administered | 12-14 |
| Outcomes | 6a | Completely defined pre-specified primary and secondary outcome measures, including how and when they were assessed | 9-11 |
|  | 6b | Any changes to trial outcomes after the trial commenced, with reasons | n/a |
| Sample size | 7a | How sample size was determined | 6-7 |
|  | 7b | When applicable, explanation of any interim analyses and stopping guidelines | n/a |
| Randomisation: |  |  |  |
| Sequence generation | 8a | Method used to generate the random allocation sequence | 8 |
|  | 8b | Type of randomisation; details of any restriction (such as blocking and block size) | 8 |
| Allocation concealment mechanism | 9 | Mechanism used to implement the random allocation sequence (such as sequentially numbered containers), describing any steps taken to conceal the sequence until interventions were assigned | 8 |
| Implementation | 10 | Who generated the random allocation sequence, who enrolled participants, and who assigned participants to interventions | 8 |
| Blinding | 11a | If done, who was blinded after assignment to interventions (for example, participants, care providers, those assessing outcomes) and how | 8 |
|  | 11b | If relevant, description of the similarity of interventions | S2 |
| Statistical methods | 12a | Statistical methods used to compare groups for primary and secondary outcomes | 15-16 |
|  | 12b | Methods for additional analyses, such as subgroup analyses and adjusted analyses | 15-16 |
| Results | | | |
| Participant flow (a diagram is strongly recommended) | 13a | For each group, the numbers of participants who were randomly assigned, received intended treatment, and were analysed for the primary outcome | 50-51 |
|  | 13b | For each group, losses and exclusions after randomisation, together with reasons | 50-51 |
| Recruitment | 14a | Dates defining the periods of recruitment and follow-up | 7 |
|  | 14b | Why the trial ended or was stopped | n/a |
| Baseline data | 15 | A table showing baseline demographic and clinical characteristics for each group | 32 |
| Numbers analysed | 16 | For each group, number of participants (denominator) included in each analysis and whether the analysis was by original assigned groups | 15 |
| Outcomes and estimation | 17a | For each primary and secondary outcome, results for each group, and the estimated effect size and its precision (such as 95% confidence interval) | 35-48 |
|  | 17b | For binary outcomes, presentation of both absolute and relative effect sizes is recommended | 34-38 |
| Ancillary analyses | 18 | Results of any other analyses performed, including subgroup analyses and adjusted analyses, distinguishing pre-specified from exploratory | Supplemental materials |
| Harms | 19 | All important harms or unintended effects in each group (for specific guidance see CONSORT for harms) | 17 |
| Discussion | | | |
| Limitations | 20 | Trial limitations, addressing sources of potential bias, imprecision, and, if relevant, multiplicity of analyses | 25-26 |
| Generalisability | 21 | Generalisability (external validity, applicability) of the trial findings | 25-26 |
| Interpretation | 22 | Interpretation consistent with results, balancing benefits and harms, and considering other relevant evidence | 21-27 |
| Other information | | |  |
| Registration | 23 | Registration number and name of trial registry | 2, 9 |
| Protocol | 24 | Where the full trial protocol can be accessed, if available | Supplementary Materials |
| Funding | 25 | Sources of funding and other support (such as supply of drugs), role of funders | 29 |

*We strongly recommend reading this statement in conjunction with the CONSORT 2010 Explanation and Elaboration for important clarifications on all the items. If relevant, we also recommend reading CONSORT extensions for cluster randomised trials, non-inferiority and equivalence trials, non-pharmacological treatments, herbal interventions, and pragmatic trials. Additional extensions are forthcoming: for those and for up to date references relevant to this checklist, see [www.consort-statement.org](http://www.consort-statement.org)

**Appendix S1.** Study protocol

**STUDY PROTOCOL**

**Treatment of Child Anxiety Disorder in the Context of Maternal Anxiety: A Randomised Controlled Trial**

**Trial Acronym: MACh (i.e. mother and child anxiety treatment study)**

RATIONALE: The outcome from CBT for children with anxiety disorders is highly variable. A major factor contributing to this is likely to be the presence of maternal anxiety and the associated disturbances in mother-child interactions and maternal behaviours. Where parental anxiety has been addressed in treatment research it has been difficult to assess its contribution to child outcome. Similarly, where therapeutic measures to address parent-child interactions have been included, it has not been possible to determine the specific role of such measures in the treatment package employed.

The trial is a three-arm RCT which aims to determine the extent to which treatments of maternal anxiety and mother-child interactions enhance standard cognitive behaviour therapy for children (CCBT) who have anxiety disorders in the context of maternal anxiety disorder (a group who currently show a poor response to treatment). Index children will receive CCBT with either additional treatment for maternal anxiety or specific measures to address features of mother-child interactions; and their outcome will be compared to that of children receiving standard individual CCBT (together with appropriate control conditions)

.**A. Background**

**Childhood Anxiety Disorders**

Anxiety disorders are the most common form of psychopathology in children. They have a significant adverse impact on children’s general socio-emotional functioning and commonly persist into adulthood.

**Treatments of childhood anxiety disorders**

Following advances in the development of successful cognitive behavioural therapies (CBT) for adult anxiety disorders (e.g. Clark & Fairburn, 1996), CBT for child anxiety disorders has now been developed. Although there is still some uncertainty over the optimal form of such intervention, recent systematic reviews of outcome research indicate that the general CBT approach produces significant therapeutic benefit in this patient group. However, it is clear from these reviews, and from the individual treatment trials, that outcome is highly variable, with a significant proportion of patients retaining their anxiety diagnoses following treatment (i.e. 16-61%; James, Soler & Wetherall, 2006).

**Predictors of Treatment Outcome**

There has been little research into the factors that predict response to CBT in anxious children, although, in addition to severity of child anxiety, two factors are likely to be especially significant: anxiety in the mother, and features of mother-child interactions.

1. *Anxiety in mothers.*

It has been known for some time that the rate of anxiety disorder amongst the parents of anxious children is raised (Last et al, 1987; Last et al, 1991), but the extent of this elevation has been uncertain and the implications for treatment outcome of child anxiety have not been fully considered. Recent research of our own has addressed this issue. In a consecutive series of children referred for treatment of an anxiety disorder, two thirds of the mothers were found to have a current DSM-IV anxiety disorder (with no elevated rate of current disorder amongst the fathers), almost three times the base rate (Cooper et al, 2006). Furthermore, follow up of the children after treatment revealed a significant association between child response and level of maternal anxiety (Cooper et al, in press).

1. *Mother-child interactions*

Specific features of mother-child interactions have been implicated in the maintenance of child anxiety, in particular, an over-controlling and over-protective maternal style (see Rapee, 1997; Wood et al, 2003) and associated maternal cognitions and expectations about child competence (Creswell et al, 2006). Notably, strong associations have been found between level of maternal anxiety and both maternal behaviours (e.g. Whaley et al, 1999; Bogels & van Melick, 2004) and maternal expectations of child competence (Wheatcroft & Creswell, 2007). It appears that the disturbances in mother-child interactions which serve to maintain child anxiety are, at least in part, themselves driven by maternal anxiety. These conclusions are supported by the findings of further research by our group. We have been conducting a prospective study of 250 infants born to mothers with anxiety disorders and control mothers to investigate the intergenerational transmission of anxiety disorders. Recent data from both this study (Murray et al, 2007), and from an associated experimental study (DeRosnay et al, 2006), have shown that a lack of both appropriate modelling and support, both features of mothers with anxiety disorders, are associated with the development of anxiety in offspring.

**Implications for optimal treatment outcomes**

In so far as CBT treatments of child anxiety disorder commonly require the day-to-day prosecution of treatment regimes to be managed by the mother (e.g. mothers are typically required to model positive responses to fear provoking stimuli and to prompt and reinforce their child’s positive responses), the mother’s own anxiety and the associated disturbances in mother-child interactions are likely to militate against optimal treatment delivery. Although the CBT treatments developed to date for the treatment of child anxiety do acknowledge the importance of both parental anxiety and parenting (e.g. Barrett et al, 1996; Mendlowitz et al, 1999; Nauta et al, 2003; Spence et al, 2000), there has been no systematic evaluation of an intervention in which both maternal anxiety and mother-child interactions are specifically addressed. There is, therefore, a need for the development and evaluation of a CBT treatment for child anxiety disorder in which maternal anxiety and associated disturbances in mother-child interactions are systematically targeted.

**Rationale for the trial**

The outcome from CBT for children with anxiety disorders is highly variable. Major factors contributing to this are likely to be the presence of maternal anxiety and associated disturbances in mother-child interactions and maternal behaviours. Where parental anxiety has been addressed in treatment research (e.g. Barrett et al, 1996; Mendlowitz et al, 1999; Nauta et al, 2003; Spence et al, 2000), for several methodological reasons, it has been difficult to assess it’s contribution to child outcome. It is notable, however, that in the single study in which treatment of parental anxiety was systematically varied, child anxiety outcome was better where therapeutic measures to address parental anxiety symptoms were included (Cobham et al, 1998). Whilst this is a finding of critical importance, since the treatment did not significantly alter levels of parental anxiety it remains unclear what aspect of the treatment effected the clinical improvement in the children. Similarly, where therapeutic measures to improve parent-child interactions have been included (e.g. Wood et al, 2006), it has not been possible to determine the specific role of such measures in the complex treatment package employed. A controlled trial in which both factors – treatment of maternal anxiety and measures to address mother-child interactions - are systematically varied, would produce data of both clinical utility and scientific importance.

**Research Questions**

In an RCT for child anxiety occurring in the context of maternal anxiety, the principal questions are:

1. Is the impact of child CBT (CCBT) enhanced by first providing CBT to the mother for her own anxiety?
2. Is the impact of CCBT enhanced by the addition of therapeutic measures designed to improve mother-child interactions?

Secondary questions are:

- 1. Is sustained improvement in child anxiety significantly associated with a reduction in maternal anxiety?
  2. Is sustained improvement in child anxiety significantly associated with improvements in maternal modelling, encouragement, over-controlling/over-protective behaviour, and associated cognitions?

**B.** **SUMMARY**

The aim of the trial is to establish the relative effectiveness of treatments of (i) maternal anxiety and (ii) key features of mother-child interactions for children with anxiety disorders who have a mother with current anxiety disorder. All treatments will be in addition to individual Cognitive Behaviour Therapy administered to all children.

Patients who consent to join the trial (participants) will be randomised to one of three conditions: (i) Child Cognitive Behaviour Therapy (CCBT) plus Cognitive Behaviour Therapy for Maternal Anxiety (MCBT); (ii) CCBT plus treatment targeting the Mother-Child Interaction (MCI), (iii) CCBT plus control conditions (see below).

| **Condition** | **Group 1: MCBT** | **Group 2:MCI** | **Group 3:CCBT** |
| --- | --- | --- | --- |
| Standard child treatment | CCBT  (child: 8 sessions) | CCBT  (child: 8 sessions) | CCBT  (child: 8 sessions) |
| Treatment of maternal anxiety | MCBT  (mother: 8 sessions) | Counselling control  (mother: 2 sessions) | Counselling control  (mother: 8 sessions) |
| Treatment of mother-child interactions | Family Health Control  (child and mother: 2 sessions; mother: 2 sessions) | MCI  (child and mother: 2 sessions; mother: 8 sessions) | Family Health Control  (child and mother: 2 sessions; mother: 2 sessions) |
| **Total therapist contact** | Child: 8 sessions  Mother: 10 sessions  Child and mother: 2 sessions | Child: 8 sessions  Mother: 10 sessions  Child and mother: 2 sessions | Child: 8 sessions  Mother: 10 sessions  Child and mother: 2 sessions |

*CCBT: Individual CBT for child anxiety; MCBT: Individual CBT for maternal anxiety; MCI: Mother-child interaction treatment*

**C. Eligibility**

The trial is open to children with a current primary diagnosis of a major anxiety disorder (Generalised Anxiety Disorder, Social Phobia, Separation Anxiety Disorder, Panic Disorder/Agoraphobia, Specific Phobia, as long as co-morbid with another anxiety disorder) whose mother also has a current major anxiety disorder.

1. Inclusion Criteria

Child:

(i) Aged 7 to 12 years;

(ii) Primary diagnosis of DSM-IV generalised anxiety disorder, social phobia, separation anxiety disorder, panic disorder/agoraphobia or specific phobia (if co-morbid with another anxiety disorder).

Mother:

(i) Primary carer;

(ii) Current maternal DSM-IV anxiety disorder.

2. Exclusion Criteria

*Participants will not be eligible if the following criteria are met.*

Child:

(i) Significant physical^[[1]](#footnote-1)^ or intellectual impairment (including autistic spectrum disorders)^[[2]](#footnote-2)^;

(ii) Current prescription of psychotropic medication (or, if psychotropic medication is prescribed, it should have been at a stable dose for at least one month with agreement to maintain that dose throughout the study);

(iii) Previously received six or more sessions of systematically administered Cognitive-Behaviour Therapy for an anxiety disorder;

Mother:

(i) Significant intellectual impairment^[[3]](#footnote-3)^;

(ii) Severe comorbid disorder (e.g. severe major depressive disorder, psychosis, substance/alcohol dependence);

(ii) Prescription of psychotropic medication (Or, if psychotropic medication is prescribed, it should have been at a stable dose for at least one month with agreement to maintain that dose throughout the study);

**D. Trial procedureS**

1. Recruitment schedule

NO

Refer back to CAMHS with detailed assessment report

Assessment by trial assessors at local CAMHS

1. Suspected anxiety disorder in child
2. Child aged 7- 12 years
3. Absence of significant physical or intellectual impairment (including ASD) in child

Does child have current anxiety disorder (Generalised Anxiety Disorder, Social Phobia, Separation Anxiety Disorder, Panic Disorder +/- Agoraphobia), specific phobia if co-morbid

YES

Does mother have current anxiety disorder? (Generalised Anxiety Disorder, Social Phobia, Panic Disorder +/- Agoraphobia), Specific phobia if > 1 specific phobia, Obsessive Compulsive Disorder

NO

Treatment as Usual

(Group CBT)

YES

Invite to take part in MRC Trial.

Does the family agree?

YES

Trial protocol

2. Treatment Interventions

There will be two stages of treatment intervention in the trial:

1. Individual Cognitive Behavioural Treatment for maternal anxiety (MCBT), or control

a. Individual CBT for maternal anxiety

This will consist of an eight session (one hour each) intervention for mothers delivered by a clinical psychologist (or equivalent) over eight-weeks. Sessions will take place in the participants’ local Child and Adolescent Mental Health Service (CAMHS), within their home, or at the University of Reading. The CBT programme will follow a manualised transdiagnostic treatment for adult anxiety disorders (Shafran, unpublished manuscript).

b. Control: Supportive Counselling

This will consist of either two or eight sessions (one hour each) of supportive counselling (see figure 1), delivered by a clinical psychologist (or equivalent) over eight-weeks. Sessions will take place in the participants’ local Child and Adolescent Mental Health Service (CAMHS), within their home, or at the University of Reading. The supportive counselling programme will follow a manualised treatment (Borkovec & Costello, 1993).

1. Individual Cognitive Behavioural Treatment for child anxiety (CCBT) with Mother Child Interaction treatment (MCI) or control

Individual CBT for child anxiety

All participating children will receive an eight session (one hour each) intervention based on the Cool Kids programme (Rapee, 2000), delivered by a clinical psychologist (or equivalent) over eight-weeks. Sessions will take place in the participants’ local Child and Adolescent Mental Health Service (CAMHS), within their home, or at the University of Reading.

1. Mother-Child Interaction Treatment

This intervention consists of 10 sessions: eight with the mother alone and two with the mother and child together. This is a novel intervention which specifically targets anxiogenic features of mother-child interactions. Specifically it aims to enhance maternal cognitions associated with child competence, reduce maternal overcontrol/overprotection, and enhance maternal warmth and encouragement. This is achieved through a combination of specific materials from existing family interventions for childhood anxiety (Rapee & Wignall, 2000; Wood et al, 2006) and video-feedback techniques developed and piloted by the trial investigators (Stein et al, 2006; Creswell et al, in press). This intervention is provided by a clinical psychologist (or equivalent) in parallel with the CCBT sessions. Sessions will generally take place in the participants’ local Child and Adolescent Mental Health Service (CAMHS), within their home, or at the University of Reading. The two mother and child sessions will be conducted within the laboratory at the University of Reading, as these involve the mother and child completing structured tasks which are video-recorded for feedback purposes.

1. Control: Family Lifestyle Management

This will consist of four sessions, two with the mother alone and two with the mother and child together. These sessions will focus on promoting a healthy lifestyle with a focus on family diet and exercise, based on existing packages applied within school settings (British Dietetic Association, 2003). This intervention is provided by a clinical psychologist (or equivalent) in parallel with the CCBT sessions. Sessions will generally take place in the participants’ local Child and Adolescent Mental Health Service (CAMHS), within their home, or at the University of Reading.

For all treatment conditions, therapists will routinely rate the extent to which participants adhere to the intervention (e.g. completion of in-session and homework exercises, session attendance).

*How the second stage interventions run in parallel is illustrated in Section R.*

**E. RANDOMISATION**

Following confirmation of eligibility and informed consent, participants will be randomised to treatment condition. Randomisation will be performed centrally by facsimile contact at the Centre for Statistics in Medicine, Oxford (CSM). This will be performed/coordinated by the Trial Statistician. The randomisation programme will include a minimisation algorithm to ensure balanced allocation of participants across the three treatment groups for the following potential prognostic factors: child age, child gender, type of child anxiety disorder (GAD, Social Phobia, SAD, Other) and baseline severity (ADIS Clinician Severity Rating) of child and mother’s primary anxiety disorder. To reduce the possibility of outcome measure events occurring after randomisation and before treatment, intervention will start within 2 weeks of randomisation.

**F. ROUTINE CARE OUTSIDE OF THE TRIAL**

Participants (mothers and children) will be asked not to engage in other psychological interventions during the course of the trial. They will also be asked not to initiate psychotropic medication and if psychotropic medication is prescribed, this should have been at a stable dose for at least one month with agreement to maintain that dose throughout the study. Referrers (Local CAMHS) and General Practitioners will be informed of this requirement.

**G. SERIOUS AND UNEXPECTED ADVERSE EVENTS**

There are no adverse side-effects of the interventions being delivered. Successful treatment of anxiety may involve some distress, however this will be managed and contained by qualified clinical psychologists, receiving regular expert supervision. Although substantial clinical benefits are anticipated from the interventions, some children and mothers can be expected to not respond to the interventions. Where children continue to meet criteria for a current anxiety disorder at the six month post treatment assessment, they will be invited to participate in a group intervention for anxious children or referred back to their local CAMHS team following clinical review and liaison. If other significant difficulties emerge these will be discussed with referrer from the local CAMHS team.

**H. ASSESSMENT OF OUTCOME**

1. Primary outcomes

The primary outcome is child anxiety (assessed both categorically [i.e. diagnosis] and continuously [i.e. symptoms]). Diagnostic status will be assessed by the ADIS for DSM-IV: C/P administered to both the mother and child. Assessors will be blind to treatment condition. Assessors’ beliefs about treatment condition will be formally assessed. Child anxiety symptoms will be assessed using questionnaires (SCAS; Spence, 1998) administered to the child, the mother and the child’s teacher. These measures will be administered post-treatment, and at 6 and 12 month follow-up assessments.

2. Secondary outcomes

Maternal anxiety will be assessed categorically using the ADIS (DSM-IV) and continuously using questionnaires (i.e. DASS, Lovibond & Lovibond, 1995; PSWQ, Meyer et al, 1990; SIAS and SPS, Mattick & Clark, 1998). These measures will be administered post-treatment, and at 6 and 12 month follow-up assessments.

Maternal interactive behaviours will be assessed by filming the mother assisting the child perform an anxiety provoking task and applying standardised ratings of anxiogenic behaviours (i.e. modelling, lack of encouragement, overcontrol/overprotection). Interactive behaviours will be coded by independent, trained, reliable raters. Coders will be blind to the purpose and conditions of the trial. Maternal cognitions will be assessed by a standardised interview. These measures will be conducted at the post-treatment assessment.

See Section S for a full assessment schedule.

3. Health Economic Assessment

An economic evaluation will be undertaken integral to the main trial. The evaluation will adhere to guidelines for good economic evaluation practice as outlined in the reference case by Gold et al (1996). The economic analysis will estimate the incremental cost and effectiveness of each of CCBT/MCBT and CCBT/MCI in relation to the control group as well as their relative costs. Patient level resource use data, including all health and social care costs (staff costs for provision of CCBT, MCBT, MCI, and the control interventions, GP costs, referrals, and other relevant services identified) as well as leisure and productivity estimates for the parents will be collected within trial forms and valued using appropriate unit costs. Staff training costs and the costs of staff supervision will also be identified and allocated pro-rata. The outcome measure for the cost-effectiveness analysis will be the ADIS as well as a measure of ‘days off school avoided’. In line with recent recommendations from the National Institute for Health and Clinical Excellence (NICE) the economic evaluation will also include generic quality of life instruments, the child friendly EuroQol EQ-5D (EuroQol, 1990; Hennesy & Kind, 2002) and HUI2 outcome measure (Feeny et al, 1995), on which normative data are available. Measures of the impact of anxiety disorders will also be included, using questionnaires administered to the child and mother (CAIS; Langley et al, 2004) and teacher (School Adjustment/ Teacher Report Form; Achenbach, 1986). These instruments will be administered at baseline, following treatment and at 6 and 12 months follow up.

**I. POWER AND SAMPLE SIZE**

A total sample size of 210 pairs of anxious children with anxious mothers will be recruited into the trial. This sample size is based on calculations relating to the primary outcome of child anxiety diagnosis for the principal questions*.*

i. Efficacy of CCBT/MCBT:

Comparison of Group 1 and the Control Group. To detect an absolute difference of 30% in success (i.e. absence of child anxiety diagnosis) post-treatment for CCBT/MCBT compared with control (40% to 70%), with 90% power at the 5% significance level (two sided) would require 56 patients per treatment group. This difference is based on reported effect of CBT with parental anxiety management in children where at least one parent had high anxiety (Cobham et al, 1998).

ii. Efficacy of CCBT/MCI:

Comparison of Group 2 and the Control Group. Assuming that the response to treatment in the control group is 40% (from Cobham et al, 1998) and the minimum clinical difference in response due to MCI is 30%, 56 patients per group are required to enable us to detect this difference with 90% power at the 5% significance level.

Thus, 56 patients are required in each of the three randomised groups. Accounting for a 20% loss to follow up would require 210 children in total to be recruited to the study. No formal comparison will be made between Groups 1 and 2 (CCBT/MCBT and CCBT/MCI). The sample size has been estimated as if two independent trials were conducted, with no adjustment for multiple testing, as recommended by Machin et al (1997).

A difference of 30% in the proportion of anxiety-free children following completion of the treatment is considered to be the minimum that would be clinically worthwhile taking into account the increased resources required and change to service delivery that would be required if either of these interventions were found to be effective and implemented in practice.

**J. DATA MANAGEMENT**

Data management will be consistent with MRC Guidelines for Good Clinical Practice in Clinical Trials (MRC, 1998) and with the Data Protection Act (1998). Principal investigators will ensure that all personnel are familiar and comply with the MRC guidelines, particularly section 5.9 ‘Data handling and record keeping’ and section 7 ‘Documentation’.

1. Identifying information

After providing consent, participants will be given a unique, sequential, study identifier. This will be used for randomisation and data entry purposes.

2. Data entry

Data will be entered in to desktop computers, fitted with SPSS for Windows v13 as standard allowing for an immediate interactive message to be displayed if an invalid data entry is made. The Trial Manager will arrange appropriate quality assurance checks.

3. Backing up of data

Immediately after every episode of data entry, data will be backed up onto a portable USB drive, which will be securely stored locally. These files will be backed up on to a password-protected system on a weekly basis. A hard copy will be printed and stored locally compliant with Data Protection Act (1998).

**K. DATA ANALYSIS**

The principal comparisons will be performed on an intention-to-treat basis. The results from the trial will be presented as comparative summary statistics (difference in proportion of anxiety-free children or mean anxiety level) with 95% confidence intervals. The analysis and reporting of results will follow the general principles of CONSORT (Moher et al, 2001).

The primary analysis will focus on the effect of the intervention following completion of treatment (post-treatment/16 week assessment). Analysis of the 6 and 12 month outcome data will utilize all outcome assessments (post treatment, 6 and 12 months) using multilevel repeated measures analysis, to establish maintenance of change.

*Child anxiety diagnosis (ADIS for DSM-IV C/P)*: The proportion of anxiety-free children in the two groups following treatment will be compared using the Chi squared test. Testing for a treatment effect after adjustment for minimisation factors will be conducted using multiple logistic regression.

*Child anxiety symptoms*: Change in anxiety scores following treatment will be analysed using multiple linear regression with baseline score and minimisation factors entered as covariates. We will formally assess the distribution of the change in anxiety scores for evidence of departure from normality. If necessary, data will either be transformed or analysed using a non-parametric equivalent. Change in anxiety scores at 6 and 12 months will be analysed using a multilevel repeated measures analysis, adjusted for baseline anxiety score and minimisation covariates.

The secondary research questions will be explored using univariate tests (e.g. Chi squared test, t-test and correlation) to examine whether the particular factors identified are associated with sustained improvement in child anxiety. Multiple logistic and linear regression will be adopted to investigate the independent factors predictive of sustained improvement in child anxiety.

A comprehensive statistical analysis plan will be produced prior to any data being seen.

**L. MANAGEMENT STRUCTURE**

1. Trial Management

The Trial Management Group (TMG) comprises the five grant holders, the clinical director (LW) and the Trial Manager (RG). The group will meet periodically throughout the trial as requested by the Principal applicant (PJC).

The day to day administration of the trial will be the overall responsibility of the principal applicant (PJC) who will monitor all aspects of recruitment, treatment and assessment, as well as the budget.

The child anxiety clinics will be under the direction of the Clinical Director (LW). She will coordinate all clinical referrals, and, together with her assistant, carry out initial clinical assessments of all referred children. Where both child and mother are found to have a current anxiety disorder, the trial manager (RG) will recruit to the trial and, in liaison with the trial statistician (NA), will ensure randomisation to treatment condition and assign to the appropriate therapists. The Trial Manager will also coordinate and supervise the maternal and child assessments. Assessment and coding of the mother-child interactions will be the under the supervision of Professor Lynne Murray.

Professor Roz Shafran (Reading) will train and supervise the adult therapists providing treatment to mothers. The therapists providing the non-directive counseling (control condition) will be supervised by an experienced counseling practitioner and supervisor to ensure adherence to protocol. The Clinical Director (LW) will supervise the two child therapists delivering CCBT to the children and the mother-child interaction treatment as well as the healthy lifestyle sessions (control). Professor Alan Stein will provide supervision to Dr Willetts on the interaction treatment.

The Trial Manager (RG) will have responsibility for the data file which will be handed over to the Trial statistician for analysis. The Trial Manager will also liaise with Dr McIntosh to ensure that all health economic data are collected appropriately.

2. Trial Steering Committee (TSC)

Overall responsibility for the trial will lie with the Trial Steering Committee comprising: Professor Jonathon Hill (Chair), Dr Gavin Malloch (MRC), Dr Natasha Conner and Vicky Taylor (Berkshire Healthcare NHS Foundation Trust), Dr Pasco Fearon (Reading) and a consumer representative. Their function is to maintain the overall integrity of the trial, to receive and consider reports from both the Trial Management Group and IDMEC and take action if appropriate. The Trial Steering Committee will meet before the trial is initiated and then every 6 months throughout the trial.

3. Independent Data Monitoring and Ethics Committee (IDMEC)

The Independent Data Monitoring and Ethics Committee will be chaired by Professor Jonathon Geddes (Oxford). Other members are Dr Craig Ramsay (Aberdeen) and a representative from Berkshire Healthcare NHS Foundation Trust). The Trial statistician will also attend meetings to present reports. The IDMEC will monitor: recruitment to the trial, protocol adherence and serious adverse events as well as the difference between trial treatments on the primary outcome measures. The IDMEC will consider reports prepared by the Trial Statistician and any other relevant studies published during the timeframe of the trial. Recommendations of IDMEC will be passed on to the Chair of the Steering Committee. The Data Monitoring and Ethics Committee will meet throughout the trial as determined by the Chair.

**M. INDEMNITY**

University of Reading indemnity will apply:

i. To meet the potential legal liability of the University of Reading for harm to participants arising from the management and design of the research.

ii. To meet the potential legal liability of the investigators/collaborators arising from harm to participants in the conduct of the research.

iii. For payment of compensation in the event of harm to the research participants where no legal liability arises.

**N. ETHICS**

Berkshire Local Research Ethics Committee has given a favourable opinion of this study (07/H0505/156), as has the University of Reading Research Ethics Committee (07/48). All aspects of the study will be conducted in line with MRC Guidelines for Good Clinical Practice in Clinical Trials (MRC, 1998).

**O. INFORMED CONSENT**

Information about the trial will be provided to both the mother and child in person from the Clinical Director (LW) as well as in written information. A copy will be provided for the participants to keep. Written consent will be obtained from parents by the Clinical Director (LW). Assent will be obtained from children. Following treatment completion, participants will be asked whether they would be happy for video-taped material to be used for teaching and training purposes. Where participants agree, separate written consent will be obtained.

**P. PUBLICATIONS AND ANCILLARY STUDIES**

1. Publications

A meeting of the Trial Management Group will be held on completion of the study to allow discussion of the main results among the collaborators. The results will then be presented to a combined meeting of the TSC and IDMEC for comment. Public presentations pertaining to the main trial must not be made without the prior agreement of the Trial Management Group.

2. Ancillary studies

Ancillary studies will be conducted by Dr Cathy Creswell (MRC Clinician Scientist Fellowship, Reading), Mr Ray Percy (PhD student, Reading) and Dr Thalia Eley (Institute of Psychiatry, London), in collaboration with Peter Cooper. The protocols for these studies will be referred to the Trial Steering Committee, whose responsibility is to safeguard the integrity of the trial, for final approval. Any further proposals for ancillary studies should initially be referred to the Trial Management group for consideration. Studies considered appropriate by the TMG will then be submitted to the TSC for final approval. In principle it is preferable for the trial to be kept as simple as possible with few further add-on studies.

**Q. PROPOSED TIMETABLE**

| **Main tasks** | **Proposed timetable** |
| --- | --- |
| Finalise protocols | May- November 2007 |
| Submit Ethics & Trust approval | August 2007 |
| Register Trial  Receipt of MRC award  In post:  Cathy Creswell (Trial Manager; MRC)  Lucy Willetts (Clinic Manager; NHS/MRC) | 1 September 2007 |
| Ethics Outcomes | November 2007 |
| Invite referrals from East and West Berks (establish wait-list for assessments)  Recruit remaining University & Trust staff  Establish satellite clinics  Convene Trial Steering Committee and IDMEC | September- December 2007 |
| Training University and Trust staff | January – February 2008 |
| Initiation of assessments | January 2008 |
| Initiation of treatment | March 2008 |
| Recruitment ends | 30 August 2010 |
| Treatments end | 31 January 2011 |
| Trials end | 30 June 2012 |

Recruitment assessments will be conducted from January 2008 until end of August 2010 (32 month), therefore we aim to recruit 6-7 new cases to the trial every month.

**R. Stage 2 Treatment (CCBT/MCI/Family Health) Outline**

| **Week** |  | **CCBT**  **(child 8 sessions)** | **MCI**  **(mother 8; mother& child 2)** | **Family Health**  **(mother 2; mother & child 2)** |
| --- | --- | --- | --- | --- |
| 1 | Session 1 | Introduction- 1. Getting to know each other  2. Psychoeducation | Mother  1. Introduction- psycho-education and rationale. | Mother  1. Introduction- healthy family lifestyle |
| 2 | Session 2 | 1. Update & review  2. How I feel depends on what I think;  Detective Thinking | Mother  1. Update & Review  2. Promoting autonomy (i):Self-help skills: giving choices, allowing struggle, attention  (ii) Feedback on video from research assessment: highlight parental positive impact on child through autonomy granting, encouragement, modelling, cognitions re child coping) |  |
| 3 | Session 3 | 1. Update & review  2. How I feel depends on what I think;  Detective Thinking Practice | Mother  1. Update & Review  2. Promoting autonomy  (i) Alternative strategies: managing child’s anxious thoughts |  |
| 4 | Session 4A | 1. Update & review  2. Rewards | Mother  1. Update & review  2. Promoting autonomy: encouraging brave behaviour; inc CALM strategy (reflective listening, selective attention , planned ignoring), positive encouragement (verbal and nonverbal), modelling brave behaviours | Mother & Child  Family diet |
| 4 | Session 4B |  | Mother & Child  Video Task: setting up an exposure hierarchy |  |
| 5 | Session 5 | 1.Update &review  2. Problem solving | Mother  1. Update & Review  2. Video feedback (to highlight successful autonomy granting).  3. Promoting autonomy: Family problem-solving |  |
| 6 | Session 6A | 1. Update & review  2. Practice | Mother  1. Update and review  2. Promoting autonomy: New roles | Mother & Child  Family exercise |
| 6 | Session 6B |  | Mother & Child  Challenging task |  |
| 7 | Session 7 | 1. Update & review  2. Practice | Mother  1. Update & Review  *2.* Video feedback (to highlight successful autonomy granting, modelling, encouragement (verbal/nonverbal), positive cognitions re child coping). |  |
| 8 | Session 8 | 1. Update & review  2. You did it | Mother  1. Update & Review  2. You did it- what helped? Future plans/ Relapse Prevention | Mother  Healthy family lifestyle  Review and summary |

**S Assessment Schedule**

| l clinical assessment  Conducted within local CAMH service | Structured clinical interviews:  1. Anxiety Disorders Interview Schedule- Child/Parent version (ADIS-C/P)  2. Anxiety Disorders Interview Schedule (ADIS) (Mother self-report)  Questionnaires:  1. Spence Children’s Anxiety Scale –parent/child version (SCAS-c/p)  2. Child Anxiety Impact Scale- parent/child version (CAIS-c/p)  3. Depression Anxiety Stress Scales (DASS)  4. Penn State Worry Inventory  5. Mattick Social Phobia Scale and Social Interaction Assessment Scale (SPS, SIAS)  6. Over-involvement questionnaire (POI) parent self-report  7. Social Communication Questionnaire (SCQ)  8.The short moods and feelings Questionnaire- Child/Parent version (SMFQ-C/P)  9.The Strengths and Difficulties Questionnaire - Child/Parent version (SDQ-C/P) |
| --- | --- |
| Research assessment 1. (pre-treatment)  Conducted at University of Reading | 1. Laboratory assessment of mother-child interaction and associated cognitions  2. Spence Children’s Anxiety Scale-teacher report (SCAS-T)  3. Teacher Report Form (TRF)  4. Teacher report- child adjustment to school  5. Health economic assessments (EQ-5D, HUI-2, diaries) |
| Research assessment 1b. (mid-treatment)  Conducted at University of Reading/ Local CAMH service | Structured clinical interviews:  1. Anxiety disorders Interview Schedule- Child/Parent version (ADIS-C/P)  2. Anxiety Disorders Interview Schedule (ADIS) (Mother self-report)  Questionnaires:  1. Spence Children’s Anxiety Scale –parent/child version (SCAS-c/p)  2. Child Anxiety Impact Scale- parent/child version (CAIS-c/p)  3. Depression Anxiety Stress Scales (DASS)  4. Penn State Worry Inventory  5. Mattick (SPS, SIAS)  6. Over-involvement questionnaire (POI) parent self-report  7. The short moods and feelings Questionnaire- Child/Parent version (SMFQ-C/P)  8. The Strengths and Difficulties Questionnaire - Child/Parent version (SDQ-C/P)  9. Therapy Questionnaire  10. Health economic assessments (EQ-5D, HUI-2, diaries) |
| Research assessment 2 (post-treatment)  Conducted at University of Reading | Structured clinical interviews:  1. Anxiety Disorders Interview Schedule- Child/Parent version (ADIS-C/P)  2. Anxiety Disorders Interview Schedule (ADIS) (Mother self-report)  Questionnaires:  1. Spence Children’s Anxiety Scale –parent/child version (SCAS-c/p)  2. Child Anxiety Impact Scale- parent/child version (CAIS-c/p)  3. Depression Anxiety Stress Scales (DASS)  4. Penn State Worry Inventory  5. Mattick (SPS, SIAS)  6. Over-involvement questionnaire (POI) parent self-report  7. The short moods and feelings Questionnaire- Child/Parent version (SMFQ-C/P)  8. The Strengths and Difficulties Questionnaire - Child/Parent version (SDQ-C/P)  9. Health economic assessments (EQ-5D, HUI-2, diaries)  Other  1. Laboratory assessment of mother-child interaction and associated cognitions |
| Research assessment 3 (6 months post-treatment)  Conducted at University Of Reading/ Local CAMH service | Structured clinical interviews:  1. Anxiety Disorders Interview Schedule- Child/Parent version (ADIS-C/P)  Questionnaires:  1. Spence Children’s Anxiety Scale –parent/child version (SCAS-c/p)  2. Child Anxiety Impact Scale- parent/child version (CAIS-c/p)  3. Depression Anxiety Stress Scales (DASS)  4. Penn State Worry Inventory  5. Mattick (SPS, SIAS)  6. Over-involvement questionnaire (POI) parent self-report  7. The Short Moods and Feelings Questionnaire- Child/Parent version (SMFQ-C/P)  8. The Strengths and Difficulties Questionnaire - Child/Parent version (SDQ-C/P)  9. Spence Children’s Anxiety Scale-teacher report (SCAS-T)  10. Teacher Report Form (TRF)  11. Teacher report- child adjustment to school  12. Health economic assessments (EQ-5D, HUI-2, diaries) |
| Research assessment 4 (12 months post-treatment)  Conducted at University Of Reading/ Local CAMH service | Structured clinical interviews:  1. Anxiety disorders Interview Schedule- Child/Parent version (ADIS-C/P)  Questionnaires:  1. Spence Children’s Anxiety Scale –parent/child version (SCAS-c/p)  2. Child Anxiety Impact Scale- parent/child version (CAIS-c/p)  3. Depression Anxiety Stress Scales (DASS)  4. Penn State Worry Inventory  5. Mattick (SPS, SIAS)  6. Over-involvement questionnaire (POI) parent self-report  7. The short moods and feelings Questionnaire- Child/Parent version (SMFQ-C/P)  8. The Strengths and Difficulties Questionnaire - Child/Parent version (SDQ-C/P)  9. Spence Children’s Anxiety Scale-teacher report (SCAS-T)  10. Teacher Report Form (TRF)  11. Teacher report- child adjustment to school  12. Health economic assessments (EQ-5D, HUI-2, diaries) |

**Appendix S2.** Statistical analysis plan

Statistical Analysis Plan

TREATMENT OF CHILD ANXIETY DISORDER IN THE CONTEXT OF MATERNAL ANXIETY: A RANDOMISED CONTROLLED TRIAL (MaCh)

Protocol Date and Version No: 14^th^ February 2008

Prepared by: Omar Omar & Merryn Voysey

Date: 29^th^ February, 2012

Centre for Statistics in Medicine

University of Oxford

| **REVIEW HISTORY** | | |
| --- | --- | --- |
| Name | Signature | Date |
| Omar Omar |  |  |
| Merryn Voysey |  |  |
| Catharine Creswell |  |  |
| Peter Cooper |  |  |

Contents

[1.1 Abbreviations 4](#_Toc314056703)

[2 INTRODUCTION 5](#_Toc314056704)

[3 BACKGROUND INFORMATION 5](#_Toc314056705)

[3.1 Study Objectives 5](#_Toc314056706)

[3.1.1 Primary Research Question 5](#_Toc314056707)

[3.1.2 Secondary Research Question 5](#_Toc314056708)

[3.2 Study Design 6](#_Toc314056709)

[3.3 Eligibility 6](#_Toc314056710)

[3.3.1 Inclusion Criteria 6](#_Toc314056711)

[3.3.2 Exclusion Criteria: 6](#_Toc314056712)

[3.4 Treatment intervention 7](#_Toc314056713)

[3.5 Sample Size 7](#_Toc314056714)

[3.6 Randomisation 8](#_Toc314056715)

[3.7 Primary and Secondary Endpoints/Outcome Measures 8](#_Toc314056716)

[3.7.1 Primary outcomes 8](#_Toc314056717)

[3.7.2 Secondary outcomes 8](#_Toc314056718)

[4 STATISTICAL METHODS 11](#_Toc314056719)

[4.1 General considerations 11](#_Toc314056720)

[4.1.1 Baseline data 11](#_Toc314056721)

[4.1.2 Consort diagram 11](#_Toc314056722)

[4.1.3 Analysis populations 11](#_Toc314056723)

[4.1.4 Missing Data 11](#_Toc314056724)

[4.2 Analyses to address primary and secondary objectives 12](#_Toc314056725)

[4.2.1 Descriptive Analyses 12](#_Toc314056726)

[4.2.2 Primary analysis 12](#_Toc314056727)

[4.2.3 Secondary analysis 12](#_Toc314056728)

[4.2.4 Subgroup analyses 13](#_Toc314056729)

[4.2.5 Sensitivity analyses 14](#_Toc314056730)

[5 CONSORT FLOWCHART 15](#_Toc314056731)

## Abbreviations

| ADIS | Anxiety Disorder Interview Schedule |
| --- | --- |
| CAIS | Child Anxiety Impact Scale |
| CBT | Cognitive Behaviour Therapy |
| CCBT | Child Cognitive Behaviour Therapy |
| CONSORT | Consolidated Standards of Reporting Trials |
| DASS | Depression Anxiety Stress Scales |
| DSM-IV | Diagnostic and Statistical Manual of Mental Disorders -IV |
| GAD | Generalised Anxiety Disorder |
| ITT | Intention to treat |
| MCBT | Maternal Cognitive Behaviour Therapy |
| MCR | Mother Child Relationship |
| PP | Per protocol |
| POI  PSWQ | Parent Over-Involvement Questionnaire  Penn State Worry Questionnaire |
| SAD | Separation Anxiety Disorder |
| SCAS | Spence Child Anxiety Scale |
| SIAS | Social Interaction Anxiety Scale |
| SPS | Social Phobia Scale |

# INTRODUCTION

This document details the proposed analysis of primary and secondary objectives for the study: “Treatment of Child Anxiety Disorder in the Context of Maternal Anxiety: A Randomised Controlled Trial”. Subsequent analyses of a more exploratory nature will not be bound by this strategy, though they are expected to follow the broad principles laid down here. The principles are not intended to curtail exploratory analysis nor to prohibit accepted practices (for example, data transformation prior to analysis), but they are intended to establish the rules that will be followed, as closely as possible, when analysing and reporting the trial.

The statistical analysis plan will be available on request when the principal papers are submitted for publication in a journal. Suggestions for subsequent analyses by journal editors or referees, will be considered carefully, and carried out as far as possible in line with the principles of this analysis strategy; if reported, the source of the suggestion will be acknowledged.

Any deviations from the statistical analysis plan will be described and justified in the final report of the trial.

The analysis of health economic data is not included in the scope of this document. These analyses will be detailed in separate analysis plans.

# BACKGROUND INFORMATION

Anxiety disorders are the most common form of psychopathology in children. They have a significant adverse impact on children’s general socio-emotional functioning and commonly persist into adulthood. Following advances in the development of successful cognitive behavioural therapies (CBT) for adult anxiety disorders, CBT for child anxiety disorders has now been developed. The outcome from CBT for children with anxiety disorders is highly variable. Major factors contributing to this are likely to be the presence of maternal anxiety and associated disturbances in the mother-child relationship and maternal behaviours. The aim of the trial is to establish the relative efficacy of treatments of (i) maternal anxiety and (ii) key features of the mother-child relationship for children with anxiety disorders who have a mother with current anxiety disorder. All treatments will be in addition to individual Cognitive Behaviour Therapy administered to all children.

## Study Objectives

### Primary Research Question

1. Is the impact of child CBT (CCBT) enhanced by providing CBT to the mother for her own anxiety?
2. Is the impact of CCBT enhanced by the addition of therapeutic measures designed to improve the quality of the mother-child relationship?

### Secondary Research Question

1. Is improvement in child anxiety significantly associated with a reduction in maternal anxiety?
2. Is improvement in child anxiety significantly associated with improvements in maternal modelling, encouragement, over-controlling/over-protective behaviour, and associated cognitions?

## Study Design

Randomised, assessor-blinded, 3-arm parallel controlled trial.

## Eligibility

### Inclusion Criteria

Child:

- Aged 7 to 12 years;
- Primary diagnosis of Diagnostic and Statistical Manual of Mental Disorders (DSM)-IV generalised anxiety disorder (GAD), social phobia, separation anxiety disorder (SAD), panic disorder/agoraphobia, or specific phobia (if co-morbid with one of the other named anxiety disorders).

Mother:

- Primary carer;
- Current maternal DSM-IV anxiety disorder.

### Exclusion Criteria:

Child:

- Significant physical or intellectual impairment (including autistic spectrum disorders) ;
- Current prescription of psychotropic medication (or, if psychotropic medication is prescribed, it should have been at a stable dose for at least one month with agreement to maintain that dose throughout the study);
- Previously received six or more sessions of systematically administered Cognitive-Behaviour Therapy for an anxiety disorder;

Mother:

- Significant intellectual impairment ;
- Severe comorbid disorder (e.g. severe major depressive disorder, psychosis, substance/alcohol dependence);
- Prescription of psychotropic medication (or, if psychotropic medication is prescribed, it should have been at a stable dose for at least one month with agreement to maintain that dose throughout the study);

##

## Treatment intervention

Patients who consent to join the trial (participants) will be randomised to one of three conditions: (i) Child Cognitive Behaviour Therapy (CCBT) plus Cognitive Behaviour Therapy for Maternal Anxiety (MCBT), (ii) CCBT plus treatment of the Mother Child Relationship (MCR) or (iii) CCBT plus control conditions (seeTable 1).

Table 1 TREATMENT INTERVENTIONS

| **Condition** | **Group 1: CCBT/MCBT** | **Group 2:**  **CCBT/MCR** | **Group 3:**  **CCBT/control** |
| --- | --- | --- | --- |
| Standard child treatment | CCBT  (child: 8 sessions) | CCBT  (child: 8 sessions) | CCBT  (child: 8 sessions) |
| Treatment of maternal anxiety | MCBT  (mother: 8 sessions) | Counselling control  (mother: 2 sessions) | Counselling control  (mother: 8 sessions) |
| Treatment of mother-child relationship | Family Health Control  (child and mother: 2 sessions; mother: 2 sessions) | MCR  (child and mother: 2 sessions; mother: 8 sessions) | Family Health Control  (child and mother: 2 sessions; mother: 2 sessions) |
| **Total therapist contact** | Child: 8 sessions  Mother: 10 sessions  Child and mother: 2 sessions | Child: 8 sessions  Mother: 10 sessions  Child and mother: 2 sessions | Child: 8 sessions  Mother: 10 sessions  Child and mother: 2 sessions |

## Sample Size

A total sample size of 210 pairs of anxious children with anxious mothers will be recruited into the trial. This sample size is based on calculations relating to the primary outcome of child anxiety diagnosis for the principal questions.

i. Efficacy of CCBT/MCBT: Comparison of Group 1 and the Control Group

To detect an absolute difference of 30% in success (i.e. absence of child anxiety diagnosis) post-treatment for CCBT/MCBT compared with control (40% to 70%), with 90% power at the 5% significance level (two sided) would require 56 patients per treatment group. This difference is based on reported effect of CBT with parental anxiety management in children where at least one parent had high anxiety (Cobham et al, 1998).

ii. Efficacy of CCBT/MCR: Comparison of Group 2 and the Control Group.

Assuming that the response to treatment in the control group is 40% (from Cobham et al, 1998) and the minimum clinical difference in response due to MCR is 30%, 56 patients per group are required to enable us to detect this difference with 90% power at the 5% significance level.

Thus, 56 patients are required in each of the three randomised groups. Accounting for a 20% loss to follow up would require 210 children in total to be recruited to the study. No formal comparison will be made between Groups 1 and 2 (CCBT/MCBT and CCBT/MCR). The sample size has been estimated as if two independent trials were conducted, with no adjustment for multiple testing, as recommended by Machin et al (1997).

## Randomisation

Following confirmation of eligibility and informed consent, participants will be randomised to treatment condition. Randomisation will be performed centrally by facsimile contact at the Centre for Statistics in Medicine, Oxford. This will be performed/coordinated by the Trial Statistician. The randomisation programme will include a minimisation algorithm to ensure balanced allocation of participants across the three treatment groups for the following potential prognostic factors: child age, child gender, type of child anxiety disorder (GAD, Social Phobia, SAD, Other),baseline severity (Anxiety Disorder Interview Scale (ADIS) Clinician Severity Rating) of child and mother’s primary anxiety disorder, and whether the child was referred by CAMHS or Primary Care. To reduce the possibility of outcome measure events occurring after randomisation and before treatment, intervention will start within 2 weeks of randomisation.

## Primary and Secondary Endpoints/Outcome Measures

### Primary outcomes

1. The primary outcome will be the proportion of children who have recovered from their primary anxiety disorder at 16 weeks as assessed by the ADIS for DSM-IV: C/P administered to both the parent(s) and child. Children will be classified as either anxiety-free for their primary anxiety disorder or not.
2. The second primary outcome is the Clinical Global Impressions of Improvement (CGI-I) scale at 16 weeks. Using this scale, children will be classified to either ‘much/very much improved’ or ‘little/no improvement’.

### Secondary outcomes

Secondary outcomes for the child;

1. The proportion of children who have recovered from their primary anxiety disorder at the 6 and 12 months follow up assessment.
2. The proportion of children who have recovered from all anxiety disorders according to ADIS at 16 weeks and at 6 and 12 months follow up.
3. The proportion of children who show improvement of the CGI at 16 weeks and at 6 and 12 months follow up.
4. Severity of child’s primary diagnosis at 16 weeks, 6 & 12 months follow up (ADIS CSR) (scale 0-8)
5. Child anxiety symptoms assessed using self-report questionnaires administered to the child and the mother (Spence Child Anxiety Scale (SCAS), Child Anxiety Impact Scale (CAIS)) and to the child’s teacher (adaptation of SCAS for teacher report, SDQ- Teacher report and Child Adjustment to School- Teacher report ) at 16 weeks, 6 and 12 months follow up.
6. Child behaviour problems as assessed by Strengths and Difficulties Questionnaire (SDQ) – conduct problems subscale – parent/child versions
7. Child low mood/depression as assessed by Short Mood and Feelings Questionnaire (SMFQ)- parent/child versions

Secondary outcomes for the mother;

1. Maternal anxiety assessed categorically using the ADIS (DSM-IV). Mothers will be classified as either free of their primary anxiety disorder or not at post-treatment (16 weeks).
2. Maternal anxiety assessed categorically using the ADIS (DSM-IV). Mothers will be classified as either free from all anxiety disorders or not at post-treatment (16 weeks).
3. Maternal anxiety assessed using questionnaires (i.e. Depression Anxiety Stress Scales (DASS), Penn State Worry Questionnaire (PSWQ 0, Social Interaction Anxiety Scale (SIAS) and Social Phobia Scale (SPS)) at 16 weeks and at 6 and 12 months follow up.
4. Severity of maternal primary diagnosis at 16 weeks follow up (ADIS CSR) (scale 0-8)
5. Maternal interactive behaviours and associated cognitions assessed by (i) filming the mother assisting the child perform an anxiety provoking task and applying standardised ratings of anxiogenic behaviours (i.e. modelling, lack of encouragement, over-control/overprotection), and (ii) POI at the post-treatment (16 week) assessment, (iii) Maternal responses to Ambiguous Scenarios Questionnaires (ASQ-p, ASQ-pc) (i.e. interpretations regarding mother and child threat, anticipated distress, perceived control, and avoidance), (iv) Maternal responses to pre and post challenge task questionnaires (i.e. expectations and appraisals regarding mother and child distress, performance and control)

Table 2: Schedule of assessments

|  | *Clinical Assessment* | *Pre-Treatment* | *Mid-Treatment* | *Post-Treatment* | *6 Months* | *12 Months* |
| --- | --- | --- | --- | --- | --- | --- |
| Anxiety Disorders Interview Schedule- Child/Parent version (ADIS-C/P) | ✓ |  | ✓ | ✓ | ✓ | ✓ |
| Anxiety Disorders Interview Schedule (ADIS) (Mother self-report) | ✓ |  | ✓ | ✓ |  |  |
| Spence Children’s Anxiety Scale –parent/child version (SCAS-c/p) | ✓ |  | ✓ | ✓ | ✓ | ✓ |
| Child Anxiety Impact Scale- parent/child version (CAIS-c/p) | ✓ |  | ✓ | ✓ | ✓ | ✓ |
| Depression Anxiety Stress Scales (DASS) | ✓ |  | ✓ | ✓ | ✓ | ✓ |
| Penn State Worry Questionnaire (PSWQ) | ✓ |  | ✓ | ✓ | ✓ | ✓ |
| Mattick Social Phobia Scale and Social Interaction Assessment Scale (SPS, SIAS) | ✓ |  | ✓ | ✓ | ✓ | ✓ |
| Parental Over-involvement questionnaire (POI) parent self-report | ✓ |  | ✓ | ✓ | ✓ | ✓ |
| Social Communication Questionnaire (SCQ) | ✓ |  |  |  |  |  |
| The short moods and feelings Questionnaire- Child/Parent version (SMFQ-C/P) | ✓ |  | ✓ | ✓ | ✓ | ✓ |
| The Strengths and Difficulties Questionnaire - Child/Parent version (SDQ-C/P) | ✓ |  | ✓ | ✓ | ✓ | ✓ |
| Laboratory assessment of mother-child interaction and associated cognitions |  | ✓ |  | ✓ |  |  |
| Spence Children’s Anxiety Scale (Teacher report) |  | ✓ |  | ✓ | ✓ | ✓ |
| Teacher report – child adjustment to school |  | ✓ |  | ✓ | ✓ | ✓ |
| Health economic assessments |  | ✓ | ✓ | ✓ | ✓ | ✓ |
| Therapy Questionnaire |  |  | ✓ |  |  |  |

#

# STATISTICAL METHODS

## General considerations

Data distributions and all model assumptions will be checked for all analyses. If model assumptions are not met, data will either be transformed or analysed using a non-parametric test as appropriate.

### Baseline data

Descriptive statistics within each randomised group will be presented for baseline values. These will include counts and percentages for binary and categorical variables; and means and standard deviations, or medians with lower and upper quartiles, for continuous variables; along with minimum and maximum values and counts of missing values. There will be no tests of statistical significance nor confidence intervals for differences between randomised groups on any baseline variable.

### Consort diagram

A flowchart showing numbers of participants screened, randomised, and those who are available for the final analysis, will be constructed. Reasons for drop-out or exclusions will be shown at each stage on a CONSORT flowchart.

### Analysis populations

The intention to treat (ITT) population will include all patients randomised.

The per-protocol (PP) population will be a subset of the ITT population. Patients excluded from the PP population will include those with the following expected protocol violations;

- Ineligibility
- Significant non-compliance defined as attendance at less than half scheduled sessions.

All analyses will be on the ITT population with a sensitivity analysis conducted on the PP population for the primary endpoints only.

### Missing Data

Multiple imputations will be utilized on the primary outcomes to account for the uncertainty attributable to missing data using multiple imputations by chained equations methods (Royston 2005)^[[4]](#footnote-4)^. Twenty imputed datasets will be created by replacing missing values with simulated values from a set of imputation models built from available baseline and outcome variables.

All baseline covariates are expected to be observed.

Baseline values will be summarised for those who did and did not complete follow up measurements to describe any characteristics related to missingness that are able to be observed.

##

## Analyses to address primary and secondary objectives

### Descriptive Analyses

For each study measure descriptive statistics within each randomised group will be presented for data at each study time point (baseline, 16 weeks, 6 months and 12 months follow up).

### Primary analysis

Both primary outcomes (sect 3.7.1) at 16 weeks will be analysed separately, using multiple log-binomial regression models, and results will be presented as relative risks with 95% confidence intervals (CI) and associated two-sided p values. Analyses will adjust for minimisation factors (child age, child gender, type of child anxiety disorder (GAD, Social Phobia, SAD, Other), baseline severity (ADIS Clinician Severity Rating) of the child’s primary anxiety disorder and baseline severity (ADIS Mother self-report) of the mother’s primary anxiety disorder). Data for the primary outcomes at 16 weeks from all three randomised arms of the trial will be used for the primary analysis and two estimates of treatment effects (Adjusted relative risks: MCBT vs Control; and MCR vs Control) will be obtained from model parameters. In the analysis, children will be included in the group to which they were initially randomised (ITT) and multiple imputations will be used to account for missing data (see Sect 4.1.4). Numbers Needed to Treat (NNT) will also be presented.

If log-binomial modelling is not possible due to model instability then other alternatives will be explored, such as Poisson regression with robust error variances^[[5]](#footnote-5)^ (a method which has the advantage of presenting results as relative risks), or logistic regression with associated odds ratios.

### Secondary analysis

Secondary analyses will be performed on the ITT population.

#### Secondary outcomes for the child;

The proportion of children who have recovered from their primary anxiety disorder at the 6 and 12 months follow up and the proportion of children who have recovered from all anxiety disorders at 16 weeks and 6 & 12 months follow up will be analysed using log-binomial regression models in the same way as primary endpoints.

Similarly, Teacher SCAS and Child Adjustment to School- Teacher report, Strengths and Difficulty Questionnaire- conduct problems (SDQ), comorbid symptoms of depression (SMFQ),and the severity of child’s primary diagnosis at 16 weeks, 6 & 12 months follow up will be analysed using linear regression models.

***Maintenance of Improvement***

The proportion of children who improved, deteriorated or remained the same from 16 weeks to 6 or 12 months assessment will be classified as follows;

Children who recovered from their primary anxiety disorder by the 16 week assessment will be categorised as maintaining improvement at 6 and 12 months or deteriorating. Children who had not recovered from their primary anxiety disorder at 16 weeks will be categorised as improving by 6 and 12 months or remaining anxious.

Similarly, children who recovered from all anxiety disorders by the 16 week assessment will be categorised as maintaining improvement at 6 and 12 months or deteriorating. Children who had not recovered from all anxiety disorders at 16 weeks will be categorised as improving by 6 and 12 months or remaining anxious.

Those who were classified as improved at 16 weeks according to CGI-I rating will be classified as maintaining improvement at 6 and 12 months or deteriorating according to the change in their CGI-I rating. Those who were not improved at 16 weeks will be classified as improving by 6 months or remaining anxious.

The proportion of children in each category (improving, maintaining improvement, remaining anxious or deteriorating) will be summarised for each randomised group according to change achieved at 16 weeks.

These analyses will be descriptive only.

#### Secondary outcomes for the mother;

The proportion of mothers who have recovered from their primary anxiety disorder and the proportion of mothers who have recovered from all anxiety disorders at 16 weeks will be analysed using log-binomial regression models.

Maternal questionnaires (DASS, POI, PSWQ, SIAS and SPS) and the severity of maternal primary diagnosis will be analysed using linear regression models at 16 weeks and 6 & 12 months follow up.

Maternal interactive behaviours (encouragement, warmth, expectations etc.) assessed through filmed laboratory sessions will be summarised by maternal anxiety type as well as by treatment group.

Regression models for these variables will follow the general principles of the primary analyses where possible depending on the distribution of the data and associated model assumptions.

All secondary analyses will adjust for minimisation factors and baseline values of the outcome under analysis. All data from all three randomised arms of the trial will be used for the analyses, and two estimates of treatment effect (MCBT vs Control; and MCR vs Control) will be obtained from model parameters.

***Association between improvement in child anxiety and scores on maternal anxiety/ maternal interactive behaviours and associated cognitions***

Correlation coefficients will be calculated to assess the association between improvement in child anxiety and maternal anxiety as well as between improvements in child anxiety and maternal-child-relationship. Partial correlation coefficients will also be calculated to adjust for any confounders. Correlation coefficients between child and mother questionnaire scores (SCAS, CAIS) will be calculated within each treatment group and overall at each time point.

### Additional analyses

Further exploration of the mediating effect of treatment for maternal anxiety/ interactive behaviours and associated cognitions (given before treatment for child anxiety) may be undertaken using specific statistical methods for mediator effects^[[6]](#footnote-6)^ and these analyses will be discussed further after initial analysis of primary and important secondary analyses are complete.

### Subgroup analyses

There are no planned subgroup analyses.

### Sensitivity analyses

There will be three sensitivity analyses conducted on both primary endpoints

1. Primary endpoint analyses will be repeated on the PP population.
2. Since the primary analyses of the main endpoints are adjusted analyses, additional unadjusted analyses of the two primary endpoints will be conducted to assess the sensitivity of the results to such adjustments. The sensitivity analysis will present a relative risk with 95% confidence interval from a simple 2 x 2 table.
3. Multiple imputation analysis is the primary analysis. Analyses will also be conducted using complete case data. Only patients with available data will be considered in this analysis.

# CONSORT FLOWCHART

Screened N=

Randomised N=

MCBT N=

MCR N=

Control N=

16 week assessment N=

16 week assessment N=

16 week assessment N=

**Appendix S3.** Adherence and fidelity

To ensure CCBT treatment adherence was equivalent across treatment arms, 75 treatment sessions (25 from each arm) were rated for adherence to the treatment manual (in terms of therapist stance, coverage of general and specific content) by blind raters (minimum BSc psychology) trained to acceptable levels of reliability (Therapist stance: *ICC* = .76 - 76; General content: *ICC* = .73 - .82; Specific content: *ICC* = .81- .89). Treatment adherence for CCBT did not differ across the three arms (Therapist stance, *F*(2,72) = 1.83, *p* = 0.17; General content, *F*(2,72) = .80, *p* = 0.92; Specific content, *F*(2,72) = .23, p = 0.80).

To ensure fidelity of MCBT and NDC, the content of therapist utterances from 100 treatment sessions (50 MCBT, 50 NDC) was allocated by independent raters (psychology graduates), trained to a high level of reliability, to categories considered as allowed or not allowed within each treatment condition (reliability of proportion of allowable utterances, MCBT *ICC* = .73; NDC counselling, *ICC* = .73).The proportion of MCBT allowable utterances was significantly higher in MCBT than NDC (*t*(98) = 6.25, *p* < .001), and the proportion of NDC allowable utterances was significantly higher in NDC than MCBT (*t*(98) = 4.40, *p* < .001), indicating that the content of the two treatments differed as intended.

To ensure treatment fidelity of MCI and HLC, raters who were blind to treatment arm rated audio recordings of 40 therapy sessions on the degree to which session content focused on the mother-child interaction or family health. Inter-rater reliability was excellent (*ICC* = .98). MCI sessions were rated significantly higher than HLC sessions on the degree to which sessions focused on the mother-child interaction (*U* = 6.01, *p* < .0001), and HLC sessions were rated significantly higher on the degree to which session focused on family healthy living (*U* = 5.90, *p* < .0001) indicating that the content of the two treatments differed as intended.

**Appendix S4.** Multiple imputation approach

Multiple imputations were used to account for missing data for the two primary end points. Twenty imputed data sets were developed using the Stata ‘ice’ function for multiple imputation with chained equations. Imputation models were developed using variables for treatment allocation, minimisation factors [child age, child gender, type of child anxiety disorder (GAD, social phobia, SAD, other), baseline severity (ADIS-C/P CSR) of the child’s primary anxiety disorder and baseline severity (ADIS-IV mother self-report) of the mother’s primary anxiety disorder] as well as assessment of ADIS-C/P CSR at assessment 1B, assessment of ADIS-C/P primary diagnosis at assessment 1B, CGI-I at assessment 1B and baseline mother’s depression (DASS-21 – depression), child depression symptoms (SMFQ-c), child behavioural problems (SDQ-conduct) and presence of child social phobia.

**Table S1.** Scores at each time point on continuous secondary outcome measures

|  |  | Initial assessment (1) | Mid treatment assessment (1B) | Post-treatment assessment (2) | 6 month post-treatment assessment (3) | 12 month post-treatment assessment (4) |
| --- | --- | --- | --- | --- | --- | --- |
|  |  |  |  |  |  |  |
| Anxiety symptoms (SCAS-c) | CCBT+ Con  CCBT+MCBT  CCBT+MCI | 40.24  (21.29)  41.33  (18.26)  39.16  (17.38) | 31.33 (17.07)  35.84 (19.63)  33.98 (16.88) | 21.00 (15.60)  29.06 (16.02)  25.28 (15.50) | 21.05  (16.24)  24.42  (17.41)  23.88  (17.37) | 18.24  (13.31)  23.09  (14.92)  21.15  (18.65) |
|  |  |  |  |  |  |  |
| Anxiety symptoms (SCAS-p) | CCBT+ Con  CCBT+MCBT  CCBT+MCI | 43.17  (15.64)  42.19  (15.53)  41.60  (16.75) | 31.11 (12.49)  32.91 (16.27)  33.13 (14.92) | 23.30 (13.81)  25.49 (14.83)  23.88  (8.71) | 22.40  (16.33)  23.21  (13.53)  22.26  (11.19) | 19.44  (13.14)  24.63  (15.37)  18.28  (12.80) |
| Anxiety symptoms (SCAS-t) | CCBT+ Con  CCBT+MCBT  CCBT+ MCI | 17.60  (13.39)  14.38  (14.57)  18.67  (12.98) |  | 11.94  (10.33)  12.18  (9.07)  15.74  (12.84) | 11.91  (13.16)  8.63  (7.05)  11.68  (11.51) | 9.44  (9.86)  15.75  (11.51)  7.70  (5.19) |
| Child Anxiety Impact Scale (CAIS-c) | CCBT+ Con  CCBT+MCBT  CCBT+ MCI | 18.29 (12.83)  23.15 (16.34)  18.91 (14.02) | 14.61 (12.25)  15.83 (11.74)  12.64 (12.45) | 10.88 (12.51)  13.80 (13.25)  12.48 (14.07) | 7.48 (7.39)  10.02 (8.56)  9.74 (9.39) | 6.75 (7.36)  11.82 (15.16)  7.82 (11.23) |
|  |  |  |  |  |  |  |
| Child Anxiety Impact Scale (CAIS-p) | CCBT+ Con  CCBT+MCBT  CCBT+ MCI | 23.17 (16.04)  25.46 (15.07)  20.55 (12.65) | 18.13 (12.07)  17.98 (13.76)  15.45 (9.35) | 12.33 (11.34)  12.50 (10.88)  10.87 (8.19) | 13.18 (12.87)  11.65 (9.67)  11.17 (9.17) | 11.48 (12.14)  14.39 (12.15)  7.92 (6.58) |
|  |  |  |  |  |  |  |
| Depression symptoms (SMFQ-c) | CCBT+ Con  CCBT+MCBT  CCBT+MCI | 7.81 (6.76)  9.03 (5.52)  7.42 (5.70) | 5.64 (5.23)  6.56 (5.23)  4.98 (4.93) | 3.06 (4.41)  6.27 (5.79)  4.91 (5.61) | 4.51 (5.29)  5.20 (4.99)  4.00 (5.17) | 3.00 (3.91)  4.31 (5.42)  4.61 (6.74) |
| Depression symptoms (SMFQ-p) | CCBT+ Con  CCBT+MCBT  CCBT+MCI | 9.39 (6.61)  10.74 (7.24)  8.79 (7.25) | 7.67 (6.39)  7.34 (6.08)  7.88 (7.36) | 4.18 (4.90)  5.26 (5.90)  4.05 (4.49) | 5.12 (5.81)  4.65 (5.25)  4.33 (4.74) | 4.06 (5.43)  6.29 (5.84)  3.71 (4.78) |
| Conduct problems (SDQ-c) | CCBT+ Con  CCBT+MCBT  CCBT+MCI | 2.97 (2.01)  3.05 (1.88)  2.84 (1.72) | 2.37 (1.79)  2.75 (1.88)  2.56 (1.84) | 2.18 (1.56)  2.44 (1.98)  2.29 (2.02) | 2.07 (1.61)  2.05 (1.38)  2.14 (1.93) | 1.64 (1.48)  2.06 (1.66)  1.95 (2.31) |
| Conduct problems (SDQ-p) | CCBT+ Con  CCBT+MCBT  CCBT+ MCI | 2.46 (1.96)  3.05(1.82)  2.57 (1.88) | 2.29 (1.93)  2.64 (2.19)  2.31 (2.05) | 1.65 (1.59)  2.02 (1.69)  1.68 (1.82) | 2.00(1.90)  1.77(1.62)  1.45(1.40) | 1.26(1.64)  1.97 (1.47)  1.82 (2.04) |
|  |  |  |  |  |  |  |
| Conduct problems (SDQ-t) | CCBT+ Con  CCBT+MCBT  CCBT+ MCI | 0.67 (1.44)  0.67 (1.08)  0.45 (1.14) |  | 1.09 (1.68)  1.04 (2.00)  0.90 (1.60) | 1.25 (2.18)  0.61 (1.54)  0.86 (1.70) | 1.22 (1.92)  0.36 (0.67)  1.33 (1.78) |
| Child Adjustment to School (CAS-t) | CCBT+ Con  CCBT+MCBT  CCBT+ MCI | 6.19 (4.84)  5.48 (4.98)  5.82 (4.60) |  | 4.61 (4.20)  3.04 (2.86)  4.90 (4.77) | 4.82 (5.08)  3.35 (3.32)  4.74 (4.54) | 5.00 (4.92)  4.60 (3.41)  5.25 (2.93) |

*Note:* CCBT+Con: Child Cognitive Behaviour Therapy + Nonspecific Control Interventions; CCBT+MCBT: CCBT + Maternal Cognitive Behaviour Therapy; CCBT+MCI: CCBT + Mother Child Interaction Treatment; SCAS-c/p Spence Children’s Anxiety Scale- child/ parent report; CAIS-c/p Child Anxiety Impact Scale child/parent report; SMFQ-c/p Short Mood and Feelings Questionnaire- child/parent report; SDQ-p Strengths and Difficulties Questionnaire – parent report.

The SCAS-c/p requires children or parents to rate how often the child experiences each of 38 anxiety symptoms, presented alongside six positive filler items in the child version (Spence, 1998; Nauta et al., 2004). The SCAS has demonstrated high internal reliability and concurrent validity with other well-known anxiety measures (Spence, 1998; Nauta et al., 2004).

The CAIS-c/p measure the extent to which anxiety interferes in a child’s life (REF 68) across three psychosocial domains (school, social activities and family functioning). Each consists of 34 items, each rated on a 4-point scale to indicate how much anxiety has caused problems. The CAIS-c/p have demonstrated good reliability and validity (Langley, Bergman, McCracken, & Piacentini, 2004; Langley et al., 2014).

The child and parent report SMFQ are brief, 13-item measures which require children or parents to report how often in the past 2 weeks they have experienced a number of symptoms of depression (Angold et al., 1995). The SMFQ-c has demonstrated high internal reliability and concurrent validity with other well-known measures of symptoms of depression (Angold et al. 1995).

The 5 item conduct problems scale from the SDQ was used to assess child, parent, and teacher reported behavioural disturbance. The Strengths and Difficulties Questionnaire is known to have good psychometric properties and scores correlate highly with other well-known scales (Goodman, 1997).

Teacher reports were collected in an attempt to provide an objective assessment of child adjustment in the school domain before and after treatment. Teachers completed an adapted version of the SCAS (Spence Child Anxiety Scale – teacher report; SCAS-t) comprising the 30 items that it was felt that teachers would be in a position to comment on (i.e. removing items about, for example, sleep, heights, animal fears). Teachers also completed the 5 item conduct scale of the teacher report form of the SDQ (Goodman, 1997). Teachers also completed a new measure of the child’s adjustment to school (Child Adjustment to School – teacher report; CAS-t), which focused on avoidance or worry about common school-based activities, such as showing things to the class, participating in group activities, speaking to the teacher. This comprised eight items that were rated on a 3-point scale from 0 (not true) to 2 (certainly true).

Internal reliability for all these scales was acceptable-good across assessment time points (SCAS-c *α* = 0.92–0.94; SCAS-p *α* = 0.88–0.93; SCAS-t *α* = 0.91–0.96; CAIS-p *α* = 0.69–0.91; SMFQ-c *α* = 0.89–0.94; SMFQ-p *α* = 0.90–0.93; SDQ-t *α* = 0.64–0.78; CAS-t *α* = 0.89–0.92), with the exception of the parent and child report SDQ conduct scales where internal reliability was marginal (SDQ-p *α* = 0.54–0.68; SDQ-c *α* = 0.55–0.69) and the CAIS-c at the initial assessment (*α* = 0.52), although for this scale internal reliability was higher at subsequent assessments (*α* = 0.88–0.96).

NB Both mother and father report questionnaires were obtained where possible, however as father responses were relatively low (n=21 -36 per group) we have reported maternal reports here.

**References**

Angold, A., Costello, E.J., Messer, S.C., Pickles, A., Winder, F., & Silver, D. (1995). Development of a short questionnaire for use in epidemiological studies of depression in children and adolescents. *International Journal of Methods in Psychiatric Research, 5,* 237–49.

Goodman, R. (1997). The strengths and difficulties questionnaire: a research note. *Journal of Child Psychology and Psychiatry, 38*, 581–6. 10.1111/j.1469-7610.1997.tb01545.x

Langley, A.K., Bergman, R.L., McCracken, J., & Piacentini, J.C. (2004). Impairment in childhood anxiety disorders: preliminary examination of the child anxiety impact scale-parent version. *Journal of Child and Adolescent Psychopharmacology, 14,* 105–14. 10.1089/104454604773840544.

Langley, A.K., Falk, A., Peris, T., Wiley, J.F., Kendall, P.C., Ginsburg, G., et al. (2014).The child anxiety impact scale: examining parent- and child-reported impairment in child anxiety disorders. *Journal of Clinical Child and Adolescent Psychology, 43,* 579–91. 10.1080/15374416.2013.817311.

Nauta, M.H,, Scholing, A., Rapee, R.M., Abbott, M., Spence, S.H., & Waters, A. (2004). A parent-report measure of children’s anxiety: psychometric properties and comparison with child-report in a clinic and normal sample. *Behaviour Research and Therapy, 42(7),* 813-839. [10.1016/S0005-7967(03)00200-6](https://doi.org/10.1016/S0005-7967(03)00200-6)

Spence, S.H. (1998). A measure of anxiety symptoms among children. *Behaviour Research and Therapy, 36,* 545–66. 10.1016/S0005-7967(98)00034-5.

Table S2. Unit costs. All costs in 2011/2012 prices. For 2010/2011 Prices adjusted for inflation using RPI 2012 or HCHS 2011/12 as appropriate.

| Item | £s | Source | Notes |
| --- | --- | --- | --- |
|  |  |  |  |
| Family doctor (GP consultation in surgery) | £40 | Personal Social Services Research Unit. Unit Costs of Health and Social Care 2012. University of Kent, 2012. | Table 10.8b. Cost including qualifications, excluding other direct care staff costs. |
| Social worker | £206 | Personal Social Services Research Unit. Unit Costs of Health and Social Care 2012. University of Kent, 2012. | Table 11.3. Cost per hour of face‐to‐face contact, including qualifications. |
| Practice nurse (nurse consultation in surgery) | £13.69 | Personal Social Services Research Unit. Unit Costs of Health and Social Care 2012. University of Kent, 2012. | Table 10.6. Cost including qualifications, excluding other direct care staff costs and based on duration of contact of 15.5 minutes. |
| Psychologist | £136 | Personal Social Services Research Unit. Unit Costs of Health and Social Care 2012. University of Kent, 2012. | Table 9.5. Cost per hour of client contact (includes A to E: A. Wages/salary; B. Salary oncosts; C. Qualifications; D. Overheads; E. Capital overheads). |
| Consultant: psychiatrist | £383 | Personal Social Services Research Unit. Unit Costs of Health and Social Care 2012. University of Kent, 2012. | Table 15.7. Cost per-face-to-face contact, including qualifications. |
| Community psychiatrist nurse (nurse –mental health) | £76 | Personal Social Services Research Unit. Unit Costs of Health and Social Care 2012. University of Kent, 2012. | Table 10.2. Cost per hour of face-to-face contact (including qualifications) |
| Education welfare officer | £20.44 | Local Government Earnings Survey 2011/12 – Observed Pay Rates.  <http://www.local.gov.uk/web/guest/local-government-intelligence/-/journal_content/56/10171/3015313/ARTICLE-TEMPLATE>  (accessed 22/04/13) | Education Welfare Officer, Median Annual Gross Pay (FTE). Unit cost calculated using information on Local Government Pension Schemes and employer National Insurance contributions. Adjusted for inflation using RPI. |
| Educational psychologist | £37.29 | Local Government Earnings Survey 2011/12 – Observed Pay Rates.  <http://www.local.gov.uk/web/guest/local-government-intelligence/-/journal_content/56/10171/3015313/ARTICLE-TEMPLATE>  (accessed 22/04/13) | Educational Psychologist, Median Annual Gross Pay (FTE). Unit cost calculated using information on Local Government Pension Schemes and employer National Insurance contributions. Adjusted for inflation using RPI. |
| Family liaison officer (school)  (approximated with family support worker) | £49 | Personal Social Services Research Unit. Unit Costs of Health and Social Care 2012. University of Kent, 2012. | Table 11.8. Costs per hour of client related work. |
| Teacher | £35.41 | Department of Education. Statistical First Release. School Workforce in England, November 2011.  <http://www.education.gov.uk/rsgateway/DB/SFR/s001062/sfr06-2012v7.pdf>  (accessed 16/04/13) | Table 9a – Average salary (£) in Total Publicly Funded School. Salary oncosts have been included in the calculation of the unit cost. Adjusted for inflation using RPI. |
| Paediatrician -  out-patient department:  paediatrics (A) | £225 | National Schedule of Reference Costs Year: ‘2011-2012’.  <https://www.gov.uk/government/publications/nhs-reference-costs-financial-year-2011-to-2012>  (accessed 18/04/13) | National Schedule of Reference Costs Year: ‘2011-2012’ – NHS Trusts. Consultant led: First Attendance Non-Admitted Face to Face. Service code: 420 |
| Audiology – out-patient department:  paediatric audiological Medicine (A),  audiological Medicine (B),  audiology (C) | £110 | As above. | As above. Weighted average of (A), (B) and (C). - (A): Service code 254 – (B): Service code 310 – (C): Service code: 840 |
| Speech and language (community speech and language therapist) | £33 | Personal Social Services Research Unit. Unit Costs of Health and Social Care 2012. University of Kent, 2012. | Table 9.3. Cost including qualifications. |
| Ophthalmology –  out-patient department:  Ophthalmology (A),  Paediatric Ophthalmology (B),  Medical Ophthalmology (C),  Orthoptics (D),  Optometry (E) | £107 | As above. | As above. Weighted average of (A), (B), (C) and (D). - (A): Service code 130 – (B): Service code 216 – (C): Service code: 460 – (D): Service code 655 – (E): Service code 662 |
| Hospital A&E Department | £108 | As above. | National Schedule of Reference Costs Year: ‘2011-2012’ – Accident and Emergency Services: No Leading to Admitted. Weighted average of all Services in the category. |
| Occupational therapist | £33 | Personal Social Services Research Unit. Unit Costs of Health and Social Care 2012. University of Kent, 2012. | Table 9.2. Cost including qualifications. |
| Paediatric dietician | £34 | As above. | Table 13.4. Cost including qualifications. |
| Paediatric physiotherapist | £74 | National Schedule of Reference Costs Year: ‘2011-2012’.  <https://www.gov.uk/government/publications/nhs-reference-costs-financial-year-2011-to-2012>  (accessed 18/04/13) | National Schedule of Reference Costs Year: ‘2011-2012’ – Community Physiotherapy Services : Child - One-to-One Services – Service code N5C1 |
| Paediatric play Specialist | £11.55 | Local Government Earnings Survey 2011/12 – Observed Pay Rates.  <http://www.local.gov.uk/web/guest/local-government-intelligence/-/journal_content/56/10171/3015313/ARTICLE-TEMPLATE>  (accessed 22/04/13) | Playworker, Median Annual Gross Pay (FTE). Unit cost calculated using information on Local Government Pension Schemes and employer National Insurance contributions. Adjusted for inflation using RPI. |
| Family therapist  (family support worker) | £49 | Personal Social Services Research Unit. Unit Costs of Health and Social Care 2012. University of Kent, 2012. | Table 11.8. Costs per hour of client related work. |
| Community children’s nurse | £93 | National Schedule of Reference Costs Year: ‘2011-2012’.  <https://www.gov.uk/government/publications/nhs-reference-costs-financial-year-2011-to-2012>  (accessed 18/04/13) | National Schedule of Reference Costs Year: ‘2011-2012’ – Community Nursing Services: Nursing Services for Children – Service code CN101 |
| Child & adolescent mental health worker | £68 | Personal Social Services Research Unit. Unit Costs of Health and Social Care 2012. University of Kent, 2012. | Table 12.6. Generic single-disciplinary CAMHS |
| Primary mental health worker | £68 | Personal Social Services Research Unit. Unit Costs of Health and Social Care 2012. University of Kent, 2012. | Table 12.6. Generic single-disciplinary CAMHS |
| Housing department | £19.81 | Local Government Earnings Survey 2011/12 – Observed Pay Rates.  <http://www.local.gov.uk/web/guest/local-government-intelligence/-/journal_content/56/10171/3015313/ARTICLE-TEMPLATE>  (accessed 22/04/13) | Housing Officer, Median Annual Gross Pay (FTE), England. Unit cost calculated using information on Local Government Pension Schemes and employer National Insurance contributions. Adjusted for inflation using RPI. |
| Citizens advice bureau | £15.50 | Office for National Statistics (UK). Labour Market, Earnings by Industry.  Patterns of Pay: Results from the Annual Survey of Hours and Earnings, 1997-2012.  <http://www.ons.gov.uk/ons/taxonomy/search/index.html?nscl=Earnings+by+Industry&nscl-orig=Earnings+by+Industry&content-type=Dataset&content-type=Reference+table&sortDirection=DESCENDING&sortBy=pubdate>  (accessed on 19/04/13) | Table 5_SIC07. Full-time employees’ pay by industry sector (SIC 2007), United Kingdom, April 2008-2012. Industry sector: other service activities. Unit cost calculated using information on Stakeholders Pension Schemes and employer National Insurance contributions. |
| Family centre (family support worker | £49 | Personal Social Services Research Unit. Unit Costs of Health and Social Care 2012. University of Kent, 2012. | Table 11.8. Costs per hour of client related work. |
| Home-start | £95.59 | McIntosh E, Barlow J, Davis H, Stewart-Brown (2009). Journal of Public Health. Sep;31(3):423-33. | Table 1, page 427. Price inflated to 2011/12 prices using the HCHS index |
| Other healthcare and social care resource use | £ 81.12 | Authors’ calculations. | Average of all other unit costs |
| Therapist: newly qualified clinical psychologist | £39.15  (per hour)  £87.98  (per hour of client contact) | April-June 2012 NHS Staff Earnings estimates  <http://www.hscic.gov.uk/catalogue/PUB07388>  accessed 23/04/13 | Table 3 – Basic Pay and Earnings for Agenda for Change Band 7 (spine point 26), and calculated according to the methodology adopted in Personal Social Services Research Unit, Unit Costs of Health and Social Care 2012. University of Kent, 2012. Table 9.5. |
| Supervisor | £70.77  (per hour)  £159.03  (per hour of client contact) | April-June 2012 NHS Staff Earnings estimates  <http://www.hscic.gov.uk/catalogue/PUB07388>  accessed 23/04/13 | Table 3 – Basic Pay and Earnings for Agenda for Change Band 8b (spine point 41), and calculated according to the methodology adopted in Personal Social Services Research Unit, Unit Costs of Health and Social Care 2012. University of Kent, 2012. Table 9.5. |
| Mileage allowance | £0.54 per mile | NHS Terms and conditions of service handbook. Amendment number 25- Pay Circular (AforC) 1/2012  <http://www.nhsemployers.org/SiteCollectionDocuments/NHS_%20tcs_handbook_v25ap260313.pdf>  accessed 12/11/13 | Car (all types of fuel) – Annual mileage up to 3,500 miles (standard rate) |
| NHS prescription costs | BNF1: £5.29  BNF2: £3.81  BNF3: £16.95  BNF4: £9.89  BNF5: £4.90  BNF6: £11.77  BNF7: £15.38  BNF8: £58.76  BNF9: £10.88  BNF10: £6.41  BNF11: £7.73  BNF12: £6.48  BNF13: £7.15  BNF14: £8.48  BNF15: £11.40 | Prescription Cost Analysis: England 2012.  <http://www.hscic.gov.uk/catalogue/PUB10610> (accessed 08/05/2014) | Totals by BNF Chapters |
| Over-the-counter (OCT) medicines | £2.40 | PAGB Annual Review 2012  <http://www.pagb.co.uk/publications/pdfs/annualreview2012.pdf>  (accessed 16/05/2014) | Fast Facts:  OCT medicines: Average product cost |
| Mother time off-work/leisure (daily rate) | £63.96 | Office for National Statistics. Annual survey of hours and earnings. Office for National Statistics; 2012  <http://www.ons.gov.uk/ons/rel/ashe/annual-survey-of-hours-and-earnings/index.html>  (accessed 14/07/14) | Table 1.1a - Weekly pay - Gross (£) - For female employee jobs: United Kingdom, 2012: Median gross weekly earning  Table 1.9a - Paid hours worked - Total - For female employee jobs: United Kingdom, 2012 |
| Cost of day of school absence | £25.55 | School spend per pupil 2011-2012.  <http://webarchive.nationalarchives.gov.uk/20130423140808/http:/education.gov.uk/schools/performance/download_data.html>  (accessed 14/07/14) | National per pupil medians  Income and Expenditure data 2011-2012 |

**Table S3.** Sensitivity analyses for base-case comparisons

| **Cost-utility analysis** | |
| --- | --- |
| **Sensitivity Analysis** | **Detail:** |
| **SA 1** | Outcomes: child QALYs  Costs: Societal perspective excluding cost of non-directive mother’s treatment |
| **SA 2** | Outcomes: mother and child combined QALYs;  Costs: NHS perspective excluding cost of non-directive mother’s treatment |
| **SA 3** | Outcomes: child QALYs  Costs: NHS perspective excluding cost of non-directive mother’s treatment |
| **SA 4** | Outcomes: mother and child combined QALYs  Costs: societal perspective excluding cost of non-directive mother’s treatment – restricted to sample of children with EQ5D reported across the 5 waves of data |
| **SA 5** | Outcomes: mother and child combined QALYs  Costs: societal perspective excluding cost of non-directive mother’s treatment – restricted to sample of children with EQ5D reported across the 5 measurement points |
| **SA 6** | Outcomes: mother and child combined QALYs  Costs: Societal perspective |
| **SA 7** | Outcomes: child QALYs  Costs: societal perspective |
| **SA 8** | Outcomes: mother and child combined QALYs  Costs: NHS perspective |
| **SA 9** | Outcomes: child QALYs  Costs: NHS perspective |
| **SA 10** | Outcomes: mother and child combined QALYs  Costs: societal perspective – restricted to sample of children with EQ5D reported across the 5 waves of data |
| **SA 11** | Outcomes: mother and child combined QALYs  Costs: societal perspective – restricted to sample of children with EQ5D reported across the 5 measurement points |

*Note:* SA: Supplementary Analysis; QALY: Quality of Life Adjusted Years

**Table S4.** Descriptive data: Maternal anxiety disorders, behaviours and cognitions

|  |  | Initial assessment (1) | Mid treatment assessment (1B) | Post-treatment assessment (2) |
| --- | --- | --- | --- | --- |
| Maternal outcomes | | | | |
| n (%) free of primary diagnosis | CCBT+Con  CCBT+MCBT  CCBT+MCI | 0  0  0 | 23 (36.5)  38 (58.5)  30 (42.9) | 28 (51.9)  36 (65.5)  41 (66.1) |
| n (%) free of all anxiety diagnoses | CCBT+Con  CCBT+MCBT  CCBT+MCI | 0  0  0 | 10 (16.13)  25 (38.46)  22 (31.43) | 19 (35.19)  26 (47.27)  29 (46.77) |
| Maternal responses | | | | |
| Observed overprotection  Mean (sd) | CCBT+Con  CCBT+MCBT  CCBT+MCI | 1.07 (0.15)  1.05 (0.12)  1.04 (0.09) |  | 1.04(0.10)  1.02 (0.05)  1.01(0.03) |
| Observed intrusiveness  Mean (sd) | CCBT+Con  CCBT+MCBT  CCBT+MCI | 1.59 (0.51)  1.68 (0.49)  1.57 (0.48) |  | 1.52 (0.44)  1.60 (0.39)  1.48 (0.38) |
| Observed positive behaviours Mean (sd) | CCBT+Con  CCBT+MCBT  CCBT+MCI | 3.10 (0.45)  3.05 (0.44)  3.10 (0.53) |  | 3.17 (0.36)  3.07 (0.36)  3.16 (0.47) |
| Observed maternal anxiety  Mean (sd) | CCBT+Con  CCBT+MCBT  CCBT+MCI | 1.65 (0.33)  1.61 (0.34)  1.63 (0.36) |  | 1.61 (0.36)  1.64 (0.41)  1.59 (0.36) |
| Maternal expectations: child fear  Mean (sd) | CCBT+Con  CCBT+MCBT  CCBT+MCI | 4.76 (1.97)  4.32 (1.75)  4.64 (1.81) |  | 3.62 (1.58)  3.10 (1.57)  3.29 (1.73) |
| Maternal expectations: child control  Mean (sd) | CCBT+Con  CCBT+MCBT  CCBT+MCI | 6.72 (1.31)  6.68 (1.46)  6.72 (1.24) |  | 6.93 (1.47)  7.32 (1.36)  7.45 (1.23) |

*Note:* CCBT+Con: Child Cognitive Behaviour Therapy + Nonspecific Control Interventions; CCBT+MCBT: CCBT + Maternal Cognitive Behaviour Therapy; CCBT+MCI: CCBT + Mother Child Interaction treatment

**Table S5.** Manipulation checks: Statistical analyses for maternal anxiety disorders, behaviours and cognitions

| **Parameter** | | | | | **Adjusted RR** | | **95% CI** | | **p-value** | |
| --- | --- | --- | --- | --- | --- | --- | --- | --- | --- | --- |
| Free from primary diagnosis at assessment 1B | | | | |  | |  | |  | |
| Treatment | | CCBT+Con | | | Ref. | |  | |  | |
|  |  | CCBT + MCBT | | | 1.63 | | 1.13 to 2.36 | | 0.009 | |
|  |  | CCBT + MCI | | | 1.22 | | 0.83 to 1.81 | | 0.314 | |
| Free from primary diagnosis at assessment 2 | | | | |  | |  | |  | |
| Treatment | | CCBT+Con | | | Ref. | |  | |  | |
|  |  | CCBT + MCBT | | | 1.23 | | 0.90 to 1.68 | | 0.201 | |
|  |  | CCBT + MCI | | | 1.27 | | 0.93 to 1.74 | | 0.126 | |
| Free from all anxiety diagnoses at assessment 1B | | | | |  | |  | |  | |
| Treatment | | CCBT+Con | | | Ref. | |  | |  | |
|  |  | CCBT + MCBT | | | 2.51 | | 1.43 to 4.40 | | 0.001 | |
|  |  | CCBT + MCI | | | 2.15 | | 1.21 to 3.81 | | 0.009 | |
| Free from all anxiety diagnoses at assessment 2 | | | | |  | |  | |  | |
| Treatment | | CCBT+Con | | | Ref. | |  | |  | |
|  |  | CCBT + MCBT | | | 1.30 | | 0.84 to 2.01 | | 0.244 | |
|  |  | CCBT + MCI | | | 1.35 | | 0.87 to 2.10 | | 0.180 | |
| **Observed behaviour** | **Treatment arm** | | **n** | **Adjusted mean change (95% CI)** | | **Adjusted mean difference (95% CI)** | | **p-value** | |  |
| Positive behaviour (–) | CCBT+Con | | 42 | 0.042 (–0.058 to 0.141) | | Ref. | |  | |  |
|  | CCBT + MCBT | | 45 | –0.009 (–0.104 to 0.086) | | –0.050 (–0.190 to 0.090) | | 0.478 | |  |
|  | CCBT + MCI | | 49 | 0.067 (–0.024 to 0.158) | | 0.026 (–0.112 to 0.163) | | 0.714 | |  |
| Overprotection (+) | CCBT+Con | | 42 | –0.016 (–0.036 to 0.005) | | Ref. | |  | |  |
|  | CCBT + MCBT | | 45 | –0.035 (–0.055 to –0.016) | | –0.020 (–0.048 to 0.009) | | 0.174 | |  |
|  | CCBT + MCI | | 49 | –0.048 (–0.066 to –0.029) | | –0.032 (–0.060 to –0.004) | | 0.026 | |  |
| Intrusiveness (+) | CCBT+Con | | 42 | –0.058 (–0.163 to 0.046) | | Ref. | |  | |  |
|  | CCBT + MCBT | | 45 | 0.015 (–0.085 to 0.116) | | 0.074 (–0.074 to 0.221) | | 0.324 | |  |
|  | CCBT + MCI | | 49 | –0.108 (–0.205 to –0.012) | | –0.050 (–0.195 to 0.195) | | 0.499 | |  |
| Expressed Anxiety (+) | CCBT+Con | | 42 | –0.005 (–0.106 to 0.097) | | Ref. | |  | |  |
|  | CCBT + MCBT | | 45 | 0.034 (–0.063 to 0.132) | | 0.039 (–0.103 to 0.182) | | 0.589 | |  |
|  | CCBT + MCI | | 49 | –0.013 (–0.107 to 0.080) | | –0.009 (–0.149 to 0.131) | | 0.901 | |  |
| **Maternal cognition** |  | |  |  | |  | |  | |  |
| Maternal expectations: child fear (+) | CCBT+Con | | 40 | -0.69 (-1.12 to -0.26) | | Ref. | |  | |  |
|  | CCBT + MCBT | | 45 | -1.22 (-1.62 to -0,82) | | -0.53 (-1.12 to -0.07) | | 0.083 | |  |
|  | CCBT + MCI | | 46 | -1.36 (-1.76 to -0.96) | | -0.67 (-1.26 to -0.07) | | 0.029 | |  |
| Maternal expectations: child control (-) | CCBT+Con | | 40 | 0.25 (-0.12 to 0.63) | | Ref. | |  | |  |
|  | CCBT + MCBT | | 45 | 0.75 (0.40 to 1.10) | | 0.50 (-0.03 to 1.02) | | 0.063 | |  |
|  | CCBT + MCI | | 46 | 0.78 (0.44 to 1.13) | | 0.53 (0.01 to 1.05) | | 0.046 | |  |

Adjusted for child age, child gender, type of child anxiety disorder (GAD, social phobia, SAD, other), baseline severity (ADIS-C/P CSR) of the child’s primary anxiety disorder and baseline severity (ADIS-IV mother self-report) of the mother’s primary anxiety disorder.

–, shows that a low score indicates dysfunction and hence an increase indicates an improvement; +, shows that a high score indicates dysfunction and hence a decrease indicates an improvement;

*Note:* Ref.: reference category; CCBT+Con: Child Cognitive Behaviour Therapy + Nonspecific Control Interventions; CCBT+MCBT: CCBT + Maternal Cognitive Behaviour Therapy; CCBT+MCI: CCBT + Mother Child Interaction Treatment

Data completeness

A high percentage of complete data were obtained from the expected key cost drivers, the therapist log measuring intervention and control resources (e.g. only 23.2% missing data for MCBT contact time). Complete EQ-5D data ranged from 47.9% to 91.3% for mother and from 46.5% to 98.6% for children. Completion rates for patient-held health and social care resource use diaries were lower. Details are summarised in Table S6 below.

Table S6. Health Economic data completeness (percentage of missing data reported)

| **Item** | **CCBT+MCBT**  **%^a^** | **CCBT+MCI**  **%^b^** | **CCBT+Con**  **%^c^** |
| --- | --- | --- | --- |
| ***Treatment*** |  |  |  |
| Child CCBT time | 34.8% | 25.4% | 38% |
| Maternal CBT time | 23.2% | n.a. | n.a. |
| MCI time | n.a. | 33.8% | n.a. |
| Non-directive counselling time | n.a. | 2.8% | 8.5% |
| Healthy Living time | 27.5% | n.a. | 11.3% |
| Supervision time for CCBT | 42.03% | 47.89% | 33.80% |
| Supervision time for MCBT | 13.04% | n.a. | n.a. |
| Supervision time for MCI | n.a. | 42.25% | n.a |
| Supervision time for non-directive counselling time | n.a. | 73.24% | 59.15% |
| Supervision time for family health | 79.71% | n.a | 74.65% |
| ***Baseline*** |  |  |  |
| Child –EQ5D score baseline | 1.45% | 2.82% | 5.63% |
| Mother –EQ5D score baseline | 8.70% | 9.86% | 15.49% |
| ***Assessment time 1B*** |  |  |  |
| Child –EQ5D score | 13.04% | 8.45% | 21.13% |
| Mother –EQ5D score | 33.33% | 28.17% | 38.03% |
| Other health and social care resources – Child & Mother | 49.30% | 33.80% | 54.90% |
| ***Assessment time 2*** |  |  |  |
| Child –EQ5D score | 33.33% | 21.13% | 32.39% |
| Mother –EQ5D score | 37.68% | 40.85% | 43.66% |
| Other health and social care resources – Child & Mother | 75.40% | 64.80% | 71.80% |
| ***6 months follow up*** |  |  |  |
| Child –EQ5D score | 36.23% | 39.44% | 40.85% |
| Mother –EQ5D score | 37.68% | 40.85% | 43.66% |
| Other health and social care resources – Child & Mother | 53.60% | 56.40% | 57.70% |
| ***12 months follow up*** |  |  |  |
| Child –EQ5D score | 50.72% | 46.48% | 53.52% |
| Mother –EQ5D score | 50.72% | 46.48% | 52.11% |
| Other health and social care resources – Child & Mother | 71.10% | 73.20% | 73.40% |

^a^ Percentage calculated with respect to the 69 patients in trial arm 1

^b^ Percentage calculated with respect to the 71 patients in trial arm 2

^c^ Percentage calculated with respect to the 71 patients in trial arm 3

*Abbrev*: Ref.: reference category; CCBT+Con: Child Cognitive Behaviour Therapy + Nonspecific Control Interventions; CCBT+MCBT: CCBT + Maternal Cognitive Behaviour Therapy; CCBT+MCI: CCBT + Mother Child Interaction Treatment

**Table S7.** Child and mother combined Quality Adjusted Life Years (QALYs) gained – CCBT+MCBT versus CCBT+Con; CCBT+MCI versus CCBT+Con

|  | **CCBT+MCBT**  **Mean (SE)**  **(n=69)** | **CCBT+MCI**  **Mean (SE)**  **(n=71)** | **CCBT+Con**  **Mean (SE)**  **(n=71)** | **Mean differences^a^**  **CCBT+MCBT – CCBT+Con**  **(95% CI)** | **P-value** | **Mean differences^a^**  **CCBT+MCI – CCBT+Con**  **(95% CI)** | **P-value** |
| --- | --- | --- | --- | --- | --- | --- | --- |
| **Baseline to 8 weeks** | 0.239  (0.005) | 0.242  (0.005) | 0.237  (0.006) | 0.002  (-0.013, 0.0179) | 0.766 | 0.005  (-0.011, 0.021) | 0.547 |
| **8 to 16 weeks** | 0.249  (0.005) | 0.256  (0.005) | 0.253  (0.006) | -0.004  (-0.018, 0.011) | 0.589 | 0.003  (-0.012, 0.019) | 0.679 |
| **16 weeks to 6 months** | 0.322  (0.006) | 0.330  (0.006) | 0.327  (0.007) | -0.005  (-0.025, 0.014) | 0.598 | 0.003  (-0.015, 0.022) | 0.709 |
| **6 to 12 months** | 0.832  (0.018) | 0.862  (0.017) | 0.849  (0.020) | -0.017  (-0.074, 0.039) | 0.540 | 0.012  (-0.037, 0.061) | 0.622 |
| **Total over 12 months** | 1.642  (0.029) | 1.690  (0.028) | 1.667  (0.034) | -0.024  (-0.115, 0.067) | 0.597 | 0.024  (-0.061, 0.108) | 0.577 |

^a^ Unadjusted difference

*Note*: CCBT+Con: Child Cognitive Behaviour Therapy + Nonspecific Control Interventions; CCBT+MCBT: CCBT + Maternal Cognitive Behaviour Therapy; CCBT+MCI: CCBT + Mother Child Interaction Treatment

**Table S8.** Cost utility analysis – CCBT+MCBT versus CCBT(+Con)

| **Scenarios** | **Cost mean difference** | **95% CI** | **QALYs mean difference^a^** | **95% CI** | **Incremental analysis (ICER reported when appropriate)** | **Probability cost effective at willingness to pay equal to £20,000 per QALY gained** | **Probability cost effective at willingness to pay equal to £30,000 per QALY gained** |
| --- | --- | --- | --- | --- | --- | --- | --- |
| Base case | £481.82 | (-£827.03, £1790.67) | -0.040 | (-0.115, 0.035) | G1 (CCBT+MCBT) is dominated | 0.114 | 0.111 |
| SA 1 | £481.82 | (-£827.03, £1790.67) | -0.040 | (-0.101, 0.021) | G1 (CCBT+MCBT) is dominated | 0.078 | 0.069 |
| SA 2 | £796.59 | (£602.63, £990.55) | -0.041 | (-0.116, 0.034) | G1 (CCBT+MCBT) is dominated | 0.018 | 0.039 |
| SA 3 | £796.59 | (£602.63, £990.55) | -0.040 | (-0.101, 0.021) | G1 (CCBT+MCBT) is dominated | 0.005 | 0.015 |
| SA 4 | -£1142.50 | (-£4087.93, £1802.94) | -0.026 | (-0.128, 0.075) | £43301.30 | 0.619 | 0.558 |
| SA 5 | £684.08 | (£380.64, £987.53) | -0.039 | (-0.141, 0.063) | G1 (CCBT+MCBT) is dominated | 0.086 | 0.122 |
| SA 6 | -£133.85 | (-£1459.31, £1191.61) | -0.040 | (-0.115, 0.035) | £3323.12 | 0.266 | 0.220 |
| SA 7 | -£133.85 | (-£1459.31, £1191.61) | -0.040 | (-0.101, 0.021) | £3331.36 | 0.231 | 0.174 |
| SA 8 | £233.55 | (-£2.96, £470.07) | -0.041 | (-0.116, 0.034) | G1 (CCBT+MCBT) is dominated | 0.086 | 0.102 |
| SA 9 | £233.55 | (-£2.96, £470.07) | -0.040 | (-0.101, 0.021) | G1 (CCBT+MCBT) is dominated | 0.049 | 0.061 |
| SA 10 | -£1880.21 | (-£4848.60, £1088.18) | -0.026 | (-0.128, 0.075) | £71506.52 | 0.747 | 0.674 |
| SA 11 | £7.66 | (-£307.27, £322.58) | -0.039 | (-0.141, 0.063) | G1 (CCBT+MCBT) is dominated | 0.235 | 0.232 |

^a^ Adjusted for child’s and mum’s baseline utilities as appropriate; SA: Sensitivity Analysis

Base case- outcomes: mother and child combined QALYs; costs: societal perspective excluding cost of non-directive mother’s treatment

SA 1 – outcomes: child QALYs; costs: societal perspective excluding cost of non-directive mother’s treatment

SA 2 - outcomes: mother and child combined QALYs; costs: NHS perspective excluding cost of non-directive mother’s treatment

SA 3 - outcomes: child QALYs; costs: NHS perspective excluding cost of non-directive mother’s treatment

SA 4 - outcomes: mother and child combined QALYs; costs: societal perspective excluding cost of non-directive mother’s treatment – restricted to sample of children with EQ5D reported across the 5 waves of data

SA 5 – outcomes: mother and child combined QALYs; costs: NHS perspective excluding cost of non-directive mother’s treatment – restricted to sample of children with EQ5D reported across the 5 measured points

SA 6 - outcomes: mother and child combined QALYs; costs: societal perspective

SA 7 – outcomes: child QALYs; costs: societal perspective

SA 8 - outcomes: mother and child combined QALYs; costs: NHS perspective

SA 9 - outcomes: child QALYs; costs: NHS perspective

SA 10 - outcomes: mother and child combined QALYs; costs: societal perspective – restricted to sample of children with EQ5D reported across the 5 waves of data

SA 11 – outcomes: mother and child combined QALYs; costs: NHS perspective – restricted to sample of children with EQ5D reported across the 5 measured points

*Note*: SA: Supplementary Analysis; CCBT: Child Cognitive Behaviour Therapy; (+Con): Including costs of nonspecific control interventions in some analyses; CCBT+MCBT: CCBT + Maternal Cognitive Behaviour Therapy; CCBT+MCI: CCBT + Mother Child Interaction Treatment

**Table S9.** Cost utility analysis – CCBT+MCI versus CCBT(+Con)

| **Scenarios** | **Cost mean difference** | **95% CI** | **QALYs mean difference^a^** | **95% CI** | **Incremental analysis (ICER reported when appropriate)** | **Probability cost effective at willingness to pay equal to £20,000 per QALY gained** | **Probability cost effective at willingness to pay equal to £30,000 per QALY gained** |
| --- | --- | --- | --- | --- | --- | --- | --- |
| Base case | £154.06 | (-£1238.95, £1547.07) | 0.020 | (-0.045, 0.085) | £7663.82 | 0.595 | 0.636 |
| SA 1 | £154.06 | (-£1238.95, £1547.07) | 0.015 | (-0.034, 0.064) | £10187.48 | 0.566 | 0.610 |
| SA 2 | £807.70 | (£609.73, £1005.67) | 0.021 | (-0.044, 0.086) | £38634.05 | 0.281 | 0.428 |
| SA 3 | £807.70 | (£609.73, £1005.67) | 0.015 | (-0.034, 0.064) | £53400.49 | 0.158 | 0.318 |
| SA 4 | -£1480.02 | (-£4303.92, £1343.88) | 0.020 | (-0.071, 0.112) | G2 (CCBT + MCI) is dominant | 0.842 | 0.828 |
| SA 5 | £653.30 | (£349.73), £956.86) | 0.015 | (-0.076, 0.107) | £42694.91 | 0.360 | 0.446 |
| SA 6 | -£478.73 | (-£1885.87, £928.42) | 0.020 | (-0.045, 0.085) | G2 (CCBT + MCI) is dominant | 0.803 | 0.799 |
| SA 7 | -£478.73 | (-£1885.87, £928.42) | 0.015 | (-0.034, 0.064) | G2 (CCBT + MCI) is dominant | 0.807 | 0.807 |
| SA 8 | £233.16 | (-£0.658, £466.99) | 0.021 | (-0.044, 0.086) | £11145.46 | 0.608 | 0.653 |
| SA 9 | £233.16 | (-£0.658, £466.99) | 0.015 | (-0.034, 0.064) | £15407.68 | 0.554 | 0.615 |
| SA 10 | -£2246.88 | (-£5091.9, £598.15) | 0.020 | (-0.071, 0.112) | G2 (CCBT + MCI) is dominant | 0.921 | 0.901 |
| SA 11 | -£44.57 | (-£359.36, £270.22) | 0.015 | (-0.076, 0.107) | G2 (CCBT + MCI) is dominant | 0.642 | 0.639 |

^a^ Adjusted for child’s and mum’s baseline utilities as appropriate; SA: Sensitivity Analysis

Base case- outcomes: mother and child combined QALYs; costs: societal perspective excluding cost of non-directive mother’s treatment

SA 1 – outcomes: child QALYs; costs: societal perspective excluding cost of non-directive mother’s treatment

SA 2 - outcomes: mother and child combined QALYs; costs: NHS perspective excluding cost of non-directive mother’s treatment

SA 3 - outcomes: child QALYs; costs: NHS perspective excluding cost of non-directive mother’s treatment

SA 4- outcomes: mother and child combined QALYs; costs: societal perspective excluding cost of non-directive mother’s treatment – restricted to sample of children with EQ5D reported across the 5 waves of data

SA 5 – outcomes: mother and child combined QALYs; costs: NHS perspective excluding cost of non-directive mother’s treatment – restricted to sample of children with EQ5D reported across the 5 measured points

SA 6 - outcomes: mother and child combined QALYs; costs: societal perspective

SA 7 – outcomes: child QALYs; costs: societal perspective

SA 8 - outcomes: mother and child combined QALYs; costs: NHS perspective

SA 9 - outcomes: child QALYs; costs: NHS perspective

SA 10 - outcomes: mother and child combined QALYs; costs: societal perspective – restricted to sample of children with EQ5D reported across the 5 waves of data

SA 11 – outcomes: mother and child QALYs; costs: NHS perspective – restricted to sample of children with EQ5D reported across the 5 measurement points

*Note*: SA: Supplementary Analysis; CCBT: Child Cognitive Behaviour Therapy; (+Con): Including costs of nonspecific control interventions in some analyses; CCBT+MCBT: CCBT + Maternal Cognitive Behaviour Therapy; CCBT+MCI: CCBT + Mother Child Interaction Treatment

**Table S10.** Societal cost mean differences – CCBT+MCBT versus CCBT; CCBT+ MCI versus CCBT(+Con)

| **Cost** | **CCBT+MCBT**  **Mean (SE)**  **(n=69)** | **CCBT+MCI**  **Mean (SE)**  **n=71** | **CCBT(+Con)**  **Mean (SE)**  **(n=71)** | **Mean differences**  **MCBT – CCBT**  **(95% CI)** | **P-value** | **Mean differences**  **MCI – CCBT**  **(95% CI)** | **P-value** |
| --- | --- | --- | --- | --- | --- | --- | --- |
| **NHS treatment 1^a,b^** | £1888.84  (78.65) | £1899.95  (80.72) | £1092.25  (61.88) | £796.59  (£602.63, £990.55) | <0.001 | £807.70  (£609.73, £1005.67) | <0.001 |
| **NHS treatment 2^a,c^** | £2,124.85  (84.98) | £2,124.46  (83.06) | £1,891.30  (87.14) | £233.55  (-£2.96, £470.07) | 0.053 | £233.16  (-£0.658, £466.99) | 0.051 |
| **Mother’s time on treatment ^a^** | £85.99 (24.62) | £91.93  (25.59) | £75.87  (25.49) | £10.12  (£1.74, £18.50) | 0.018 | £16.06  (£7.59, £24.54) | <0.001 |
| **Child’s school absence** | £256.64  (67.62) | £179.75  (31.003) | £292.91  (64.63) | -£36.26  (-£212.37, £139.84) | 0.679 | -£113.15  (-£253.88, £27.58) | 0.112 |
| **Mother’s time off work** | £183.77  (55.07) | £157.45  (44.44) | £445.24  (121.99) | -£261.47  (-£536.79, £13.85) | 0.062 | -£287.80  (-£547.89, -£27.70) | 0.030 |
| **Mother’s time off usual activities** | £187.43  (58.64) | £263.95  (91.68) | £203.59  (72.03) | -£16.16  (-£201.06, £168.74) | 0.862 | £60.36  (-£170.21, £290.93) | 0.603 |
| **Child’s other health & social care and non-NHS services** | £911.58  (222.61) | £700.39  (211.11) | £862.79  (231.83) | £48.79  (-£613.12, £710.70) | 0.883 | -£162.41  (-£805.40, £480.59) | 0.615 |
| **Mother’s other health & social care and non-NHS services** | £843.92  (138.82) | £735.40  (208.18) | £966.96  (246.07) | -£123.04  (-£681.71, £435.62) | 0.662 | -£231.56  (-£860.04, £396.91) | 0.463 |
| **Drugs (mother and child)** | £141.93  (26.75) | £137.91  (18.54) | £131.30  (25.07) | £10.63  (-£53.77, £75.03) | 0.743 | £6.61  (-£51.98, £65.20) | 0.822 |
| **Total societal cost 1^b^** | £4476.86  (329.59) | £4149.09  (432.30) | £3995.04  (570.52) | £481.82  (£827.03, £1790.67) | 0.469 | £154.06  (-£1238.95, £1547.07) | 0.828 |
| **Total societal cost 2^c^** | £4736.11 (330.35) | £4391.23 (433.31) | £4869.96 (579.41) | -£133.85  (-£1459.31,£1191.61) | 0.843 | -£478.73  (-£1885.87, £928.42) | 0.504 |

^a^ One child/mother belonging to the MCBT treatment arm also received MCI mother only (minutes=420 / cost=£68.04) and 'child and mother’ treatment (minutes=35 / cost=£5.67) ^b^Excluding cost of non-directive mother’s treatments ^c^Including cost of non-directive mother’s treatments

*Note*: CCBT: Child Cognitive Behaviour Therapy; (+Con): Including costs of nonspecific control interventions in some analyses; CCBT+MCBT: CCBT + Maternal Cognitive Behaviour Therapy; CCBT+MCI: CCBT + Mother Child Interaction Treatment

Table S11. Treatment resource use mean differences – CCBT+MCBT versus CCBT+Con

| **Resource use item** | **CCBT+MCBT**  **mean minutes (SD)**  **(n=69)** | **CCBT+Con**  **mean minutes (SD)**  **(n=71)** | **Mean differences minutes**  **CCBT+MCBT – CCBT+Con (95% CI)** | **P-value** |
| --- | --- | --- | --- | --- |
| **CCBT** | 423.95  (143.26) | 391.74  (183.95) | 32.21  (-22.99, 87.41) | 0.251 |
| **MCBT/NDC** | 380.79  (106.30) | 329.73  (110.05) | 51.06  (14.89, 87.24) | 0.006 |
| **MCI /HLC** | 150.00  (72.94) | 138.61  (70.85) | 11.39  (-12.63, 35.43) | 0.350 |
| **Supervision time for CCBT (therapist time)** | 26.22  (3.42)^1^ | 26.22  (3.55)^1^ | 0.0019596  (-9.10, 9.10) | 1.000 |
| **Supervision time for CCBT(supervisor time)** | 26.22  (3.42)^1^ | 26.22  (3.55)^1^ | 0.0019596  (-9.10, 9.10) | 1.000 |
| **Supervision time for MCBT/NDC (therapist time)** | 46.03  (4.59)^1^ | 52.21  (6.62)^1^ | -6.18  (-22.77, 10.42) | 0.456 |
| **Supervision time for MCBT/NDC (supervisor time)** | 46.03  (4.59)^1^ | 52.21  (6.62)^1^ | -6.18  (-22.77, 10.42) | 0.456 |
| **Supervision time for MCI/HLC (therapist time)** | 8.77  (2.36)^1^ | 9.09  (2.06)^1^ | -0.32  (-5.88, 5.24) | 0.905 |
| **Supervision time for MCI/HLC (supervisor time)** | 8.77  (2.36)^1^ | 9.09  (2.06)^1^ | -0.32  (-5.88, 5.24) | 0.905 |
| **Preparation time & record keeping** | 376.22  (192.47) | 404.28  (191.75) | -28.06  (-92.27, 36.15) | 0.39 |
| **Travel (duration)** | 317.13  (437.18) | 191.08  (298.52) | 126.05  (1.25, 250.84) | 0.048 |
| **Travel (mileage)** | 181.31  (260.03) | 105.69  (180.75) | 75.63  (0.97, 150.29) | 0.047 |
| **Other** | 33.04  (48.06) | 30.26  (86.41) | 2.78  (-20.68, 26.24) | 0.815 |
| **Extra- time associated to ‘not- attended’ sessions (e.g. waiting time, phone call, etc)** | 0.80  (4.67) | 0.42  (3.56) | 0.37  (-1.01, 1.76) | 0.594 |
| **Total therapy resource use^2^ (minutes)** | 1843.98  (81.78)^1^ | 1661.18  (78.67)^1^ | 182.79  (-40.92, 406.51) | 0.108 |

^1^Standard error in parentheses, as variable is multiply imputed or is derived from other multiply imputed variables.

^2^Excluding travel mileage, which is not expressed in minutes.

*Note*: CCBT+Con: Child Cognitive Behaviour Therapy + Nonspecific Control Interventions; CCBT+MCBT: CCBT + Maternal Cognitive Behaviour Therapy; CCBT+MCI: CCBT + Mother Child Interaction Treatment; NDC: Non-Directive Counselling; HLC: Healthy Living Control

# **Table S12.** Treatment resource use mean differences – CCBT+MCI versus CCBT+Con

| **Resource use item** | **CCBT+MCI****mean (SD)****(minutes)****n=71** | | **CCBT+Con****mean (SD)** **(minutes)****(n=71)** | **Mean differences****CCBT+MCI – CCBT+Con (95% CI)****(minutes)** | **P-value** |
| --- | --- | --- | --- | --- | --- |
| **CCBT** | 454.31(147.13) | 391.74(183.95) | | 62.57(7.30, 117.84) | 0.027 |
| **MCBT/NDC** | 108.77(20.27) | 329.73(110.05) | | -220.97(-247.22, -194.71) | <0.001 |
| **MCI/HLC** | 458.73(148.85) | 138.61(70.85) | | 320.12(281.45, 358.80) | <0.001 |
| **Supervision time for CCBT (therapist time)** | 30.43(3.77)^3^ | 26.22(3.55)^3^ | | 4.21(-6.52, 14.94) | 0.436 |
| **Supervision time for CCBT (supervision time)** | 30.43(3.77)^1^ | 26.22(3.55)^1^ | | 4.21(-6.52, 14.94) | 0.436 |
| **Supervision time for MCBT/NDC (therapist time)** | 35.50(5.09)^1^ | 52.21(6.62)^1^ | | -16.72(-32.21, -1.22) | 0.035 |
| **Supervision time for MCBT/NDC (supervision time)** | 35.50(5.09)^1^ | 52.21(6.62)^1^ | | -16.72(-32.21, -1.22) | 0.035 |
| **Supervision time for MCI/FH (therapist time)** | 24.99(2.55)^1^ | 9.09(2.06)^1^ | | 15.89789(9.30, 22.49) | <0.001 |
| **Supervision time for MCI/FH (supervision time)** | 24.99(2.55)^1^ | 9.09(2.06)^1^ | | 15.89789(9.30, 22.49) | <0.001 |
| **Preparation time & record keeping** | 346.93(157.95) | 404.28(191.75) | | -57.36(-115.64, 0.93) | 0.054 |
| **Travel (duration)** | 221.48(379.50) | 191.08(298.52) | | 30.39(-82.89, 143.68) | 0.597 |
| **Travel (mileage)** | 127.73(231.39) | 105.69(180.75) | | 22.05(-46.85, 90.94) | 0.528 |
| **Other** | 20.61(49.35) | 30.26(86.41) | | -9.65(-32.99, 13.70) | 0.415 |
| **Extra- time associated to ‘not- attended’ sessions (e.g. waiting time, phone call, etc)** | 3.52(17.14) | 0.42(3.56) | | 3.10(-1.01, 7.21) | 0.138 |
| **Total therapy resource use^2^****(minutes)** | 1796.18(75.27)^1^ | 1661.18(78.67)^1^ | | 134.99(-79.71, 349.69) | 0.216 |

# ^1^Standard error in parentheses, as variable is multiply imputed or is derived from other multiply imputed variables.

# ^2^Excluding travel mileage, which is not expressed in minutes.

*Note*: CCBT+Con: Child Cognitive Behaviour Therapy + Nonspecific Control Interventions; CCBT+MCBT: CCBT + Maternal Cognitive Behaviour Therapy; CCBT+MCI: CCBT + Mother Child Interaction Treatment; NDC: Non-Directive Counselling; HLC: Healthy Living Control

1. Where physical disability would impede treatment delivery (e.g. significant speech/ hearing impairment). [↑](#footnote-ref-1)
2. Significant intellectual impairment will be determined by children being registered within local learning disability services. Children will be excluded if they have a current diagnosis of an Autistic Spectrum Disorder (ASD). In case of undiagnosed ASD, a preliminary assessment will be made at the initial assessment (see Section S). [↑](#footnote-ref-2)
3. Significant intellectual impairment will be determined by the mother being registered within local learning disability services. [↑](#footnote-ref-3)
4. Royston P (2005). Multiple imputation of missing values: update of ice. *Stata Journal 5(4)*: 527-536 [↑](#footnote-ref-4)
5. Zou G. A Modified Poisson Regression Approach to Prospective Studies with Binary Data. *Am J Epidemiol.* Apr 1 2004;159(7):702-706. [↑](#footnote-ref-5)
6. Emsley R, Dunn R. Mediation and moderation of treatment effects in randomised controlled trials of complex interventions. Statistical Methods in Medical Research 2010; 19: 237–270 [↑](#footnote-ref-6)
